# Antagonistic Roles for KNOX1 and KNOX2 Genes in Patterning the Land Plant Body Plan Following an Ancient Gene Duplication

**DOI:** 10.1371/journal.pgen.1004980

**Published:** 2015-02-11

**Authors:** Chihiro Furumizu, John Paul Alvarez, Keiko Sakakibara, John L. Bowman

**Affiliations:** 1 School of Biological Sciences, Monash University, Melbourne, Australia; 2 Graduate School of Science, University of Tokyo, Hongo, Tokyo, Japan; 3 Department of Plant Biology, University of California Davis, Davis, California, United States of America; Peking University, CHINA

## Abstract

Neofunctionalization following gene duplication is thought to be one of the key drivers in generating evolutionary novelty. A gene duplication in a common ancestor of land plants produced two classes of KNOTTED-like TALE homeobox genes, class I (KNOX1) and class II (KNOX2). KNOX1 genes are linked to tissue proliferation and maintenance of meristematic potentials of flowering plant and moss sporophytes, and modulation of KNOX1 activity is implicated in contributing to leaf shape diversity of flowering plants. While KNOX2 function has been shown to repress the gametophytic (haploid) developmental program during moss sporophyte (diploid) development, little is known about KNOX2 function in flowering plants, hindering syntheses regarding the relationship between two classes of KNOX genes in the context of land plant evolution. *Arabidopsis* plants harboring loss-of-function KNOX2 alleles exhibit impaired differentiation of all aerial organs and have highly complex leaves, phenocopying gain-of-function KNOX1 alleles. Conversely, gain-of-function KNOX2 alleles in conjunction with a presumptive heterodimeric BELL TALE homeobox partner suppressed SAM activity in *Arabidopsis* and reduced leaf complexity in the *Arabidopsis* relative *Cardamine hirsuta*, reminiscent of loss-of-function KNOX1 alleles. Little evidence was found indicative of epistasis or mutual repression between KNOX1 and KNOX2 genes. KNOX proteins heterodimerize with BELL TALE homeobox proteins to form functional complexes, and contrary to earlier reports based on *in vitro* and heterologous expression, we find high selectivity between KNOX and BELL partners *in vivo*. Thus, KNOX2 genes confer opposing activities rather than redundant roles with KNOX1 genes, and together they act to direct the development of all above-ground organs of the *Arabidopsis* sporophyte. We infer that following the KNOX1/KNOX2 gene duplication in an ancestor of land plants, neofunctionalization led to evolution of antagonistic biochemical activity thereby facilitating the evolution of more complex sporophyte transcriptional networks, providing plasticity for the morphological evolution of land plant body plans.

## Author Summary

Eukaryotes alternate between haploid (1*n*) and diploid (2*n*) stages during their life cycles, and often seen are remarkable differences in morphology and physiology between them. Land plants are multicellular in both generations, in contrast to their presumed ancestral green algae that develop multicellularity only in the haploid stage. TALE class homeodomain transcriptional factors play a key role in the activation of diploid development in diverse lineages of eukaryotes. A gene duplication event within this family in an ancestor of land plants had profound implications for land plant evolution. We show that the two subclasses resulting from the gene duplication event act to pattern, in a complementary manner, most above ground organs of the diploid stage of the flowering plant *Arabidopsis*. Their opposing activities sculpt the shape of leaves from entire to pinnate and control the architecture of the plant body, and thus providing plasticity for evolutionary tinkering. These results form a foundation for understanding how these genes have been co-opted from an ancestral role of regulating diploid gene expression in a zygote to directing sporophyte land plant body architecture and provide insight into the evolution of various forms of life cycles.

## Introduction

**Fig 1:**
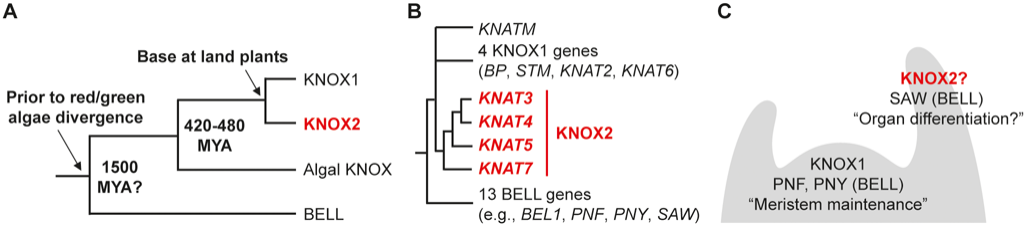
Phylogeny and expression patterns of KNOX and BELL genes. (**A**) Phylogenetic relationships of KNOX and BELL gene families in the plant lineage. Available sequence information suggests the gene duplication producing the KNOX and BELL genes occurred before the divergence of red and green algae. A gene duplication in the lineage leading to land plants created KNOX1 and KNOX2 genes from an ancestral algal KNOX gene. Estimated dates for some nodes are listed in millions of years before present (Mya). (**B**) In *Arabidopsis*, KNOX1, KNOX2, and BELL proteins are encoded by 4, 4, and 13 genes, respectively. In addition, *KNATM* encodes for a KNOX-related protein lacking a homeodomain. Detailed phylogenetic analyses of KNOX genes are presented in S1 and S2 Figs. (**C**) Schematic depiction of expression patterns for *Arabidopsis* KNOX1, KNOX2, and BELL genes based on previous literature [75] and publicly available transcriptome data (for details, see S3 and S4 Figs.). KNOX1 and some BELL genes, e.g., *PNF* and *PNY*, are primarily expressed in meristematic tissues while KNOX2 and other BELL genes such as *SAW1* and *SAW2* are expressed in differentiating organs. KNOX2 genes are highlighted in red.

Gene duplication is thought to be one of the key drivers in generating evolutionary novelty. Following gene duplication, paralogs can undergo a process of neofunctionalization, supplying a genetic basis for morphological novelty [1,2,3]. Transcription factors can undergo neofunctionalization via either a change in expression pattern or an alteration in functionality, e.g. the derivation of a repressor or inhibitor from an ancestral activator, or vice versa (e.g. [4]). Three amino acid loop extension (TALE) homeodomain transcriptional factors, characterized by having a homeodomain that has three extra amino acids between helices 1 and 2, are found in all eukaryotic lineages [5,6,7]. Plant TALE homeobox genes are classified into two subfamilies, KNOTTED-like homeobox (KNOX) and BELL-like (BELL) [8]. Whilst Chlorophyte algal KNOX genes are of a single class, a gene duplication in a common ancestor of land plants, produced two classes of KNOX genes, class I (KNOX1) and class II (KNOX2) [8,9] (Fig. 1A). KNOX genes of flowering plants have been studied for over two decades, however, the functional consequences of the KNOX gene duplication have been largely unexplored.

The first identified plant homeobox gene was *Knotted1*, a KNOX1 gene of maize [10]. Since, KNOX1 genes have been characterized in numerous flowering plants with a conspicuous loss-of-function phenotype being a failure in shoot apical meristem (SAM) maintenance. KNOX1 activity is also involved in maintenance of meristematic activity during leaf development, with prolonged activity in leaf margins observed in species with complex leaves and gain-of-function alleles result in more complex leaves. Thus, KNOX1 genes play a critical role in maintaining meristematic properties of cells in flowering plant sporophytes, the diploid generation of the land plant life cycle (reviewed in [11,12,13]). The KNOX1 genes of the moss *Physcomitrella patens* are only expressed in the sporophyte and mutants have decreased sporophyte growth, suggesting that KNOX1 genes have a conserved role in tissue proliferation during sporophyte development throughout land plants [12,13,14]. There is no evidence indicative of KNOX1 function in the gametophyte (haploid) generation in any characterized species, including the indeterminate meristems of the moss gametophyte, suggesting the role of KNOX1 is restricted to the diploid, sporophyte generation [14].

A functional distinction between KNOX1 and KNOX2 genes has been postulated from studies based on gene expression patterns in flowering plants. Northern blot analyses in maize demonstrated that KNOX1 gene expression is confined to less differentiated tissues whereas KNOX2 genes are broadly expressed in differentiating tissues and mature organs [9]. Similar broad expression profiles of KNOX2 genes have been reported in *Arabidopsis* [15,16] and tomato [17]. Characterization of spatial expression patterns in *Arabidopsis* revealed that KNOX2 genes have both overlapping and distinct expression patterns and that they are expressed in most tissues except for meristematic regions [15,18,19,20,21]. Despite several reports of expression patterns, comparatively little is known about KNOX2 gene function in flowering plants. One of the four *Arabidopsis* KNOX2 paralogs, *KNAT7*, is involved in secondary cell wall biosynthesis [18,19,21], and another, *KNAT3*, is reported to modulate ABA responses [22]. While these findings are consistent with the reported expression patterns, there exists a gap between broad expression patterns and known KNOX2 functions. For instance, unlike KNOX1 genes, which are important regulators of growth and development, it is not clear whether or not KNOX2 genes are involved in morphogenesis in flowering plants. These questions have gone unanswered owing to the paucity of functional studies on KNOX2 genes due to extensive genetic redundancy as noted by Truernit et al. [20].

From a wider perspective, a possible ancestral function of TALE homeodomain proteins is the regulation of diploid gene expression upon fusion of gametes, as is observed in the Chlorophyte alga *Chlamydomonas reinhardtii* and several fungi [23,24,25]. In *C. reinhardtii* the *plus* gamete expresses a BELL protein while the *minus* gamete expresses a KNOX protein; upon gamete fusion the KNOX and BELL proteins heterodimerize and regulate zygotic gene expression [25]. In the moss *P. patens*, KNOX2 genes are expressed in the egg cells and the sporophyte. Eliminating KNOX2 activity results in apospory, the development of a haploid body plan during the diploid generation, suggesting KNOX2 genes regulate the gametophyte-to-sporophyte morphological transition, a reflection of the hypothesized ancestral TALE homeodomain gene function [26]. Thus, both KNOX1 and KNOX2 mutant phenotypes in land plants are consistent with the hypothesis that ancestral function of KNOX genes was to regulate diploid gene expression. However, the seemingly different roles of KNOX1 and KNOX2 genes indicate functional diversification among land plant KNOX genes.

To gain insight into developmental roles for KNOX2 genes in flowering plants and the genetic relationship between KNOX1 and KNOX2 classes, we undertook a genetic study of KNOX2 genes in *Arabidopsis thaliana*, a species in which KNOX1 gene function is well characterized. We discuss the implication of our findings on the impact of the gene duplication producing KNOX1 and KNOX2 paralogs in the course of land plant evolution.

## Results

Four *Arabidopsis* genes encode KNOX2 proteins. As phylogenetic analyses place *KNAT7* in a clade sister to the remaining *Arabidopsis* KNOX2 genes [9], we focused on *KNAT3* (AT5G25220), *KNAT4* (AT5G11060), and *KNAT5* (AT4G32040) genes in this study (Fig. 1B, S1 and S2 Figs.). In contrast to KNOX1 genes, which are expressed primarily in meristematic tissues, KNOX2 gene expression occurs in differentiating organs suggesting distinct and perhaps complementary functions [15,16,20] (Fig. 1C, S3 and S4 Figs.).

### KNOX2 mutant phenotypes and expression patterns

**Fig 2:**
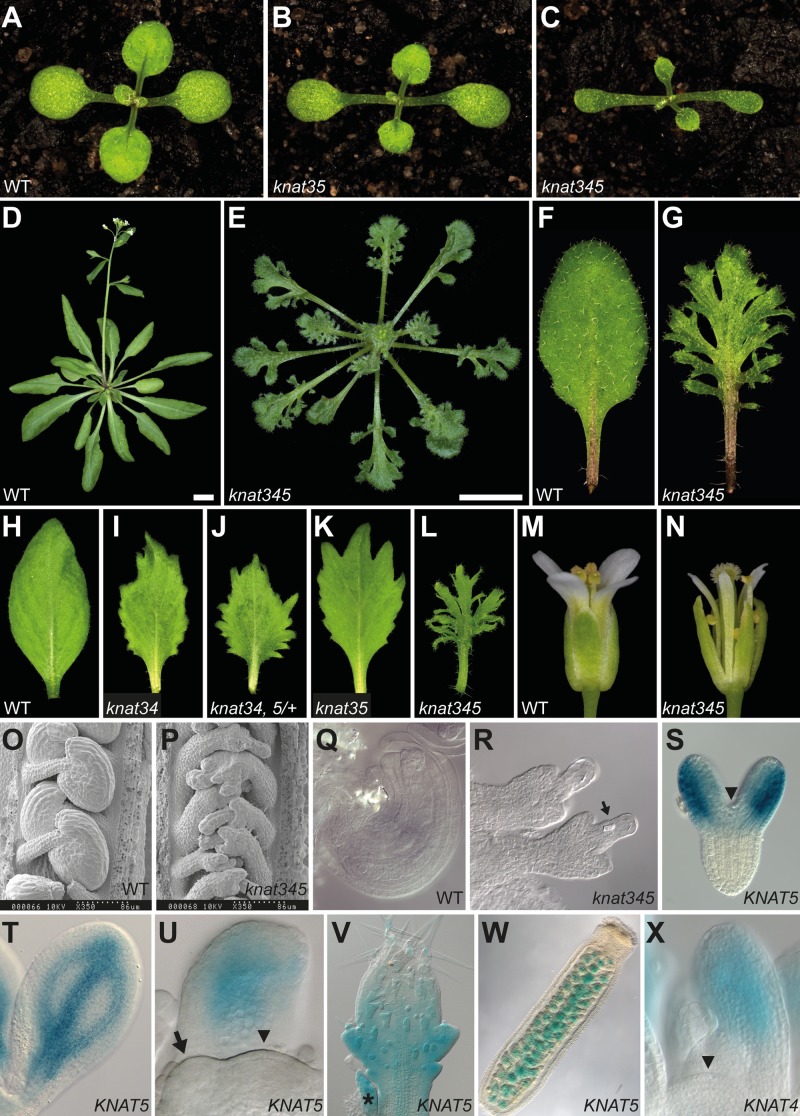
KNOX2 mutant phenotypes and *KNAT5* expression patterns. (**A-C**) 10-day-old seedlings of wild-type (WT, **A**), *knat3 knat5* (designated as *knat35*, **B**), and *knat345* (**C**). (**D-E**) Wild-type (**D**) and *knat345* (**E**) plants after bolting; 5 weeks-old plants are shown. (**F-G**) Representative wild-type (**F**) and *knat345* (**G**) rosette leaves. (**H-L**) First cauline (stem) leaves of wild-type (**H**), *knat3 knat4* (designated as *knat34*, **I**), *knat3 knat4 knat5*/+ (designated as *knat34*, *5*/+, **J**), *knat3 knat5* (**K**), and *knat345* (**L**) plants; progressive loss of the KNOX2 activity results in increasingly serrated leaves. (**M-N**) Wild-type (**M**) and *knat345* (**N**) flowers. (**O-R**) Mature wild-type (**O, Q**) and *knat345* (**P, R**) ovules and embryo sacs. An arrow marks ectopic formation of tracheary elements. (**S-V**) _*pro*_ *KNAT5*:*KNAT5-GUS* expression in developing embryos (**S-T**), vegetative shoot apex (**U**), leaf (V), and ovules (**W**). _*pro*_ *KNAT5*:*KNAT5-GUS* is not detected in the shoot apical meristem (marked by an arrowhead) and the youngest leaf primordium (marked with an arrow). An asterisk indicates a stipule. (**X**) _*pro*_ *KNAT4*:*GUS* expression in vegetative shoot apex. For additional expression data, see S10 Fig. Plants are all in the Columbia (Col) background. Scale bars, 1 cm.

KNOX2 mutant phenotypes were characterized using null alleles (S5 Fig.). As reported previously [20], single mutants lack conspicuous aberrant phenotypes. Amongst double mutants, *knat3 knat5* seedlings are distinguishable from wild type by a longer petiole and narrower lamina of cotyledons, and more deeply serrated leaf margins (Fig. 2A-B). Venation pattern is also affected in *knat3 knat5* cotyledons (S6 Fig.). *knat3 knat4* plants also have serrated leaves (Fig. 2I) and are sporophytically female sterile with abnormal integument development. While *knat3 knat4/+ knat5* plants are also female sterile, *knat3/+ knat4 knat5* plants are phenotypically wild type and produce viable seeds, facilitating characterization of segregating triple mutant plants.

Selfed *knat3/+ knat4 knat5* plants segregated small, dark-green plants with deeply lobed leaves (Fig. 2C-G and S14F Fig.), a phenotype reminiscent of gain-of-function KNOX1 alleles [12,13]. PCR-based genotyping indicated these plants were triple-mutant homozygotes (*knat345*). Since only a single mutant allele was available for each gene, we designed an artificial miRNA (amiRNA, [27]) targeting only *KNAT4*, *amiR* ^*159*^ *-KNAT4* (S7A Fig.), and generated *knat3 knat5* plants constitutively expressing *amiR* ^*159*^ *-KNAT4* under the control of the Cauliflower Mosaic Virus 35S promoter (_*pro*_ *35S*). _*pro*_ *35S*:*amiR* ^*159*^ *-KNAT4 knat3 knat5* lines closely resembled the identified *knat345* plants (S8C-D Fig.). Another amiRNA, *amiR* ^*159*^ *-KNAT345–1*, was designed to target *KNAT3*, *KNAT4*, and *KNAT5* (S7B Fig.). _*pro*_ *35S*:*amiR* ^*159*^ *-KNAT345–1* plants also show a deeply serrated leaf phenotype (S8E and S14H Figs.). We thus conclude that this is the triple mutant phenotype. Consistent with functional redundancy among these genes, dosage-dependent enhancement of the leaf serration phenotype was observed (Fig. 2H-L). Likewise, venation pattern in cotyledons is more severely affected in *knat345* plants (S6 Fig.). Floral organs homologous with leaves are also affected. Sepals and petals are narrower and partially dissected in the *knat345* mutant, and integument development is defective as seen in *knat3 knat4* plants (Fig. 2M-N and S9 Fig.). Ectopic formation of tracheary elements is observed in *knat345* embryo sacs (Fig. 2R). Although these genes are expressed in roots [15,16,20], the morphology of primary roots in *knat345* plants appeared normal (S10 Fig.).

A _*pro*_ *KNAT5*:*KNAT5-GUS* translational fusion line was generated to monitor expression patterns (Fig. 2S-W and S11 Fig.). In line with the mutant phenotypes, GUS activity was observed in developing leaves but excluded from the shoot apical meristem (SAM) (Fig. 2S-V and S11C Fig.). During early stages of leaf development, GUS activity was not detected in youngest leaf primordia but was observed in older leaf primordia (Fig. 2U). Reduced signal levels were observed in older leaves (S11B Fig.). Prolonged incubation detected GUS signal along cotyledon and leaf veins and in ovules (Fig. 2T and S11H-I Fig.). _*pro*_ *KNAT5*:*KNAT5-GUS* expression is nuclear in trichomes, supporting a role for KNAT5 in transcriptional regulation (S11 Fig.). A transcriptional fusion line, _*pro*_ *KNAT4*:*GUS*, was generated to examine *KNAT4* expression patterns. Ten independent T1 plants were examined, all of which exhibited *KNAT4* promoter activity in leaves but not in the SAM (Fig. 2X). A similar expression pattern has been described for *KNAT3* using either a GUS reporter line or RNA *in situ* hybridization [15]. Exclusion of KNOX2 expression from the SAM is also supported by cell-type specific expression analyses of the inflorescence SAM (S3 Fig.).

### Genetic evidence for KNOX2/BELL heterodimarization

**Fig 3:**
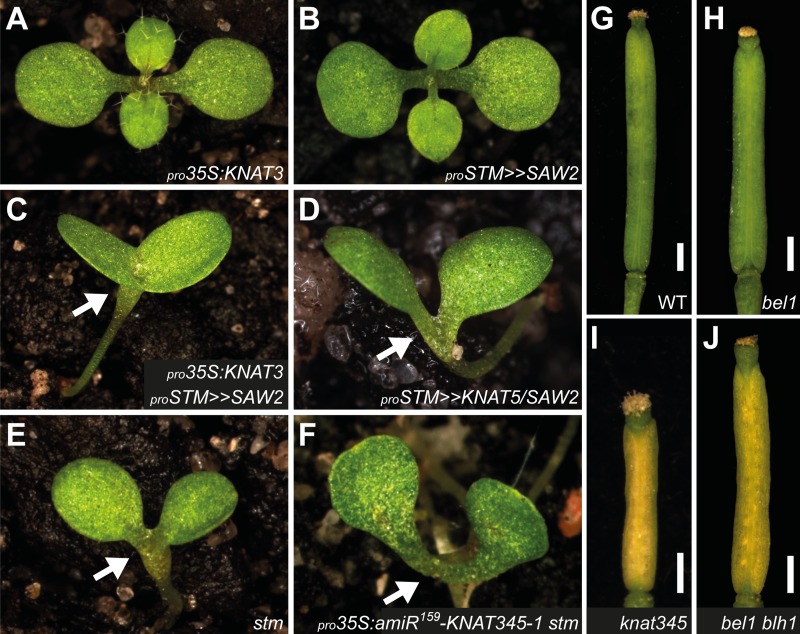
Genetic interactions between BELL and KNOX2 genes. (**A-E**) 10 days-old seedlings of _*pro*_ *35S*:*KNAT3* (**A**), _*pro*_ *STM>>SAW2* (**B**), _*pro*_ *35S*:*KNAT3*_*pro*_ *STM>>SAW2* (**C**), _*pro*_ *STM>>KNAT5*_*pro*_ *STM>>SAW2* (**D**), and *stm-11* (**E**), showing resemblance between *stm* (**E**) and plants expressing both KNOX2 and SAW2 proteins in the meristem (**C, D**). Defective meristems are marked with arrows. In (B-D), expression of *SAW2* or *KNAT5* alone, or both genes together is transactivated by the *STM* regulatory sequence. (**F**) Introducing the _*pro*_ *35S*:*amiR* ^*159*^ *-KNAT345–1* construct did not alter the seedling lethal phenotype of *stm-11* plants. Shown is a 25 days-old plant. (**G-J**) Gynoecia from wild-type (**G**, emasculated and unpollinated), *bel1–154* (**H**), *knat345* (**I**), and *bel1–154 blh1–114* (**J**) flowers. (**I-J**) Unfertilized gynoecia turn yellow. See S9 Fig. for more data. This phenotype was not observed in emasculated wild-type (**G**), *bel1* (**H**), and *blh1–114* plants. Plants in (**B, D, E, F**) are in the Landsberg *erecta* (L*er*) background. A plant in (**C**) is in the Col/L*er* mixed background. All other plants are in the Col background. Scale bars, 0.5 mm.

KNOX and BELL heterodimerization plays a pivotal role in regulating their activities as transcription factors [13]. We speculated that the lack of BELL partners may explain why no conspicuous phenotype has been described to date upon ectopic expression of KNOX2 genes [28] (S12 Fig.). The founding BELL gene, *BELL1* (*BEL1*), and closely related paralogs, *SAWTOOTH1* (*SAW1*) and *SAW2*, represent candidates for KNOX2 partners since loss-of-function phenotypes in ovules and leaf margins resemble those of KNOX2 mutants [29,30,31]. Physical interactions have been previously proposed between these BELL and KNOX2 proteins [22,29,32]. *SAW1* and *SAW2* are expressed in leaves but not in meristems [29] (S3 and S4 Figs.). Thus, we co-expressed *SAW2* and *KNAT3* throughout the SAM by trans-activating SAW2 under the control of *SHOOT MERISTEMLESS* (*STM*) regulatory sequences (_*pro*_ *STM>>SAW2*; >> denotes the use of transactivation system hereafter) in _*pro*_ *35S*:*KNAT3* plants [33]. _*pro*_ *35S*:*KNAT3*_*pro*_ *STM>>SAW2* plants lack an embryonic SAM and resemble loss-of-function *stm* or *stm knat6* mutant plants [34,35,36] (Fig. 3A-C, 3E). Combined expression of KNAT5 and SAW2 in the _*pro*_ *STM* region resulted in a similar phenotype (Fig. 3D), confirming that the presence of both SAW2 and KNOX2 proteins simultaneously accounts for the phenotype. Collectively, these data indicate that concurrent expression, and by proxy, heterodimerization with BELL proteins, is important for KNOX2 function and that KNOX2 activity may thus be constrained by limited access to corresponding BELL partners.

A mutation in *KNAT3* suppresses the gain-of-function phenotype caused by ectopic expression of another BELL gene, *BLH1*, suggesting that BLH1 is likely a functional partner for KNOX2 proteins [37]. This prompted us to examine genetic interactions between *BLH1* and *BEL1*-related BELL genes, and we found that *bel1 blh1* double mutants show color changes in unfertilized gynoecia as seen in *knat3 knat4* and *knat345* plants (Fig. 3G-J and S9 Fig.). Thus, *BEL1* and *BLH1* play a redundant role in gynoecium development and perhaps act in association with KNOX2 genes. More comprehensive genetic analyses as well as expression analyses are required to assign specific roles to functionally redundant BELL genes.

### KNOX2 exhibits selectivity for BELL

To further dissect BELL-KNOX interactions, plants expressing BELL and/or KNOX genes in the _*pro*_ *STM* region were characterized. Among BELL proteins, PENNYWISE (PNY) and POUND-FOOLISH (PNF) are expressed in the SAM and act in conjunction with KNOX1 proteins to promote SAM activity [12,13]. As expected, plants expressing KNOX1 genes (*STM* or *KNAT2*) or *PNY* in the STM domain appeared wild type (S13B-D Fig.). In contrast, _*pro*_ *STM>>SAW2* plants displayed abnormal floral morphologies, such as fused sepals, reduced petals, and misshapen fruits (S13E, H Fig.), phenotypes often associated with reduced KNOX1 activity, e.g. weak *stm* mutants [38]. Flower development was not impacted in _*pro*_ *STM>>KNAT5* plants, but fused sepals are also observed in strong _*pro*_ *35S*:*KNAT3* lines (S12F Fig.). Concomitant expression of KNOX2 with PNY or KNOX1 with SAW2 did not enhance the KNOX2 or SAW2 overexpression phenotypes. We therefore conclude KNOX2 shows selectivity for BELL proteins *in vivo*.

### KNOX2 mutant phenotype is independent of KNOX1 activity

**Fig 4:**
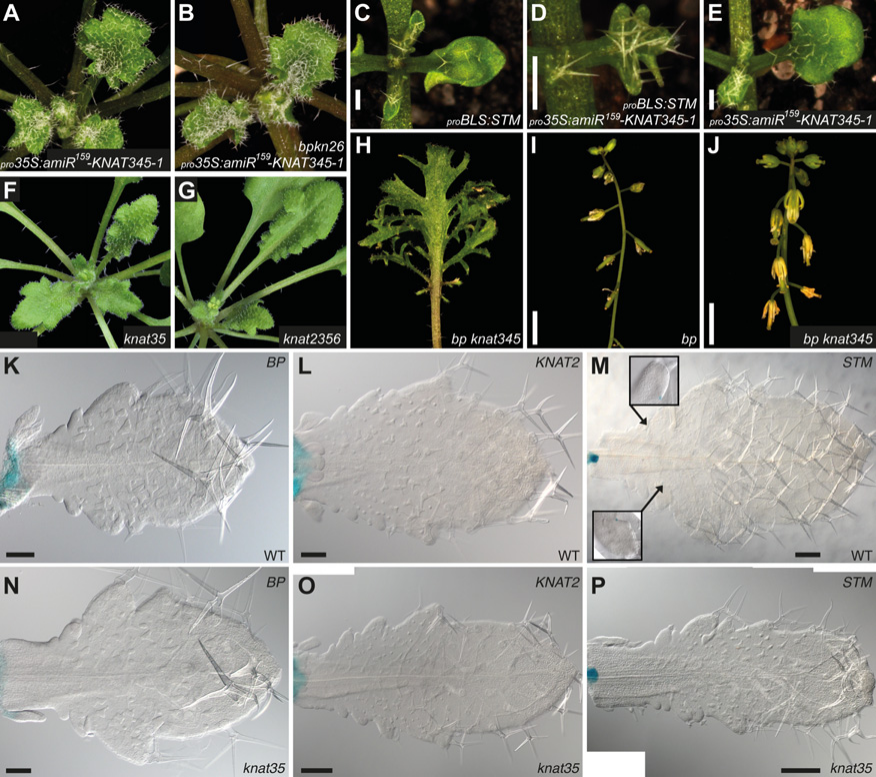
Genetic interactions between KNOX1 and KNOX2 genes. (**A-B**) Morphology of _*pro*_ *35S*:*amiR* ^*159*^ *-KNAT345–1* plants in wild-type (**A**) and *bp-9 knat2–5 knat6–1* mutant (**B**) backgrounds. 6 weeks-old plants are shown. (**C-E**) F1 plants expressing both _*pro*_ *BLS*:*STM* and _*pro*_ *35S*:*amiR* ^*159*^ *-KNAT345–1* constructs (**D**) show a stronger serration phenotype than either parental line (**C, E**). All plants shown are hemizygous for the transgene(s) and 2 weeks old. (**F-G**) Morphology of *knat3 knat5* (**F**) and *knat2–5 knat3 knat5 knat6–1* (**G**) plants grown for 5 weeks. (**H**) A representative *bp knat345* rosette leaf with deeply lobed margins, as seen in *knat345*. (**I-J**) The *bp* inflorescence phenotype (**I**) is observed in *bp knat345* infloresences (**J**), indicating additive effects of mutations in these genes. (**H-J**) Plants were grown for 2 months. (**K-P**) KNOX1 reporter expression in wild-type (**K-M**) and *knat3 knat5* (**N-P**) plants. Shown are _*pro*_ *BP*:*GUS* expression in 17 days-old plants (**K, N**), _*pro*_ *KNAT2*:*GUS* expression in 20 days-old plants (**L, O**), and _*pro*_ *STM*:*GUS* expression in 21 days-old plants (**M, P**). No ectopic expression of _*pro*_ *BP*:*GUS* was detected during stages of leaf development when lobes are forming in *knat3 knat5* plants (**K, N**), but ectopic expression was observed at leaf serration tips after their development. _*pro*_ *KNAT2*:*GUS* and _*pro*_ *STM*:*GUS* expression patterns in *knat3 knat5* plants are similar to those in wild-type plants (**L-M, O-P**). Occasionally, longer incubation detected _*pro*_ *STM*:*GUS* activity in the sinus of wild-type and mutant leaves (**M**). _*pro*_ *KNAT2*:*GUS* and _*pro*_ *STM*:*GUS* were analyzed in the mixed genetic background (refer to S1 Table for details), and other plants are in the Col background. Scale bars in **I**, **J**, 3 mm, **K**, **L**, **N**, 100 μm and in **M**, **O**, **P**, 200 μm.

Loss-of-function and gain-of-function KNOX2 phenotypes are reminiscent of gain-of-function and loss-of-function KNOX1 phenotypes, respectively [12,13]. To characterize the relationship between the two gene classes, loss-of-function alleles for KNOX1 and KNOX2 were combined. Plants constitutively expressing an amiRNA targeting *KNAT3*, *KNAT4*, and *KNAT5*, _*pro*_ *35S*:*amiR* ^*159*^ *-KNAT345–1*, in KNOX1 loss-of-function (*stm* or *bp knat2 knat6*) backgrounds were examined. Neither the meristem failure of *stm* mutants nor the KNOX2 loss-of-function mutant leaf phenotype was suppressed in these plants (Fig. 3F and Fig. 4A-B). Similarly, neither *knat2 knat3 knat5 knat6* nor *bp knat345* showed significant suppression of the KNOX2 loss-of-function mutant leaf phenotype and the *bp* inflorescence phenotype (Fig. 4F-J). Thus, loss-of-function phenotypes of KNOX1 and KNOX2 mutants are not due to ectopic activation of KNOX2 and KNOX1, respectively. Furthermore, *BP*, *STM*, and *KNAT2* expression was not altered in *knat3 knat5* plants (Fig. 4K-P), arguing against mutual repression between KNOX1 and KNOX2 genes.

Deeply lobed leaves, a phenotype characteristic of gain-of-function KNOX1 alleles, occur in *Arabidopsis* plants where *STM* is driven by the leaf specific promoter, _*pro*_ *BLS*, _*pro*_ *BLS*:*STM* ([39]; S14B, C Fig.). These were crossed with loss-of-function KNOX2 plants (_*pro*_ *35S*:*amiR* ^*159*^ *-KNAT345–1*) to generate plants with ectopic KNOX1 and reduced KNOX2 activities in the leaves. Compared to the parental lines, F1 plants harboring both transgenes displayed more extreme leaf margin elaboration (Fig. 4C-E). The additive effects, rather than epistatic interactions, suggest it is unlikely that the two subclasses negatively regulate one another.

### Antagonistic relationship between KNOX1 and KNOX2

**Fig 5:**
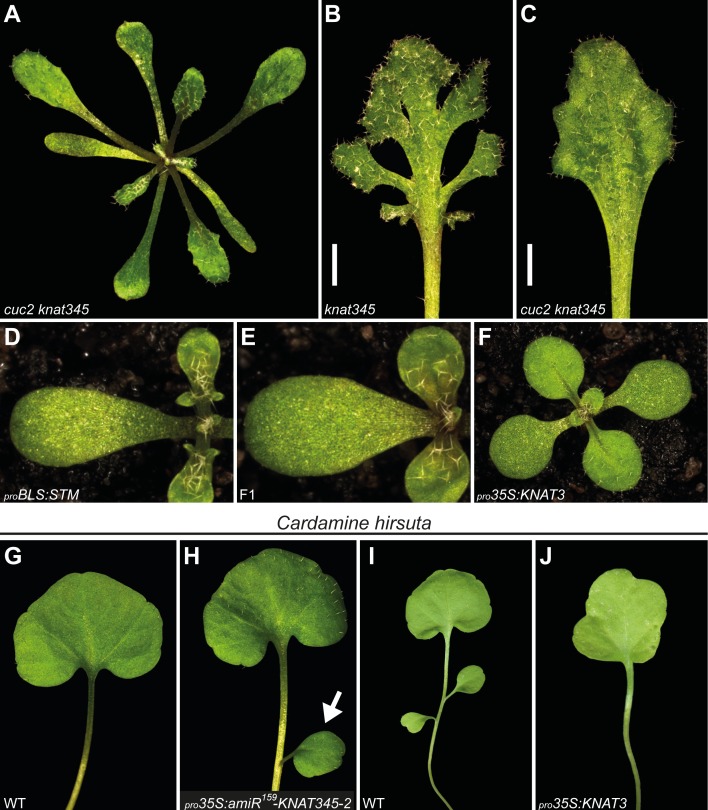
KNOX1 and KNOX2 converge on CUC activity. (**A**) One-month old *cuc2 knat345* quadruple mutant. A mutation in the *CUC2* gene largely suppresses the leaf serration phenotype of *knat345* plants. Compare with a *knat345* plant in Fig. 2E. See S13 Fig. for additional data. (**B-C**) Representative *knat345* (**B**) and *cuc2 knat345* (**C**) rosette leaves, demonstrating marginal leaf lobing is suppressed by a *cuc2* mutation. Shown are the 10th leaves from 2 month-old plants of each genotype. Note that other mutant phenotypes, including leaf size and female sterility, are not suppressed by the *cuc2* mutation. (**D-F**) _*pro*_ *35S*:*KNAT3* partially suppresses the leaf lobing phenotype of _*pro*_ *BLS*:*STM* plants. 12 days-old plants are shown. (**G-H**) Second leaves of wild-type (**G**) and _*pro*_ *35S*:*amiR* ^*159*^ *-KNAT345–2* (**H**) *Cardamine hirsuta* plants grown for four weeks. (**G**) In wild-type plants, the first and second leaves always consist of a single, undivided, lamina. (**H**) Reducing KNOX2 activity by consitutively expressing an amiRNA that targets *Cardamine hirsuta* orthologues of *KNAT3*, *KNAT4*, and *KNAT5* genes (_*pro*_ *35S*:*amiR* ^*159*^ *-KNAT345–2*) results in plants with an extra lateral leaflet (marked with an arrow) on the second leaf. (**I-J**) Third leaves removed from one month-old wild-type (**I**) and _*pro*_ *35S*:*KNAT3* (**J**) *Cardamine hirsuta* plants. (**I**) In wild-type plants, the third leaf typically consists of three leaflets. (**J**) Introduction of gain-of-function KNOX2 alleles (constitutive expression of the *KNAT3* gene from *Arabidopsis*; _*pro*_ *35S*:*KNAT3*) results in an undivided third leaf in strong lines. Plants in (A-F) are all in the Col background.

An attractive hypothesis for the antagonism between KNOX1 and KNOX2 is that they regulate shared downstream events in an opposite manner. The complex leaf of gain-of-function KNOX1 alelles is suppressed by reduction in CUP SHAPED COTYLEDON (CUC) transcription factor activity [40] (S14C Fig.). Two CUC genes are targeted by the miR164 family of miRNAs, and expression of *miR164b* in young leaves using regulatory sequences of the *FILAMENTOUS FLOWER* (*FIL*) gene (designated as _pro_FIL), _*pro*_ *FIL*:*miR164b*, flattens the leaf margin in wild-type plants (S14D-E Fig.). Thus, the miRNA-mediated CUC regulation plays a key role in leaf margin elaboration [41]. Introduction of _*pro*_ *FIL*:*miR164b* also suppressed the leaf dissection phenotype in a *knat345* mutant background (S14F-G Fig.). Among miR164 targets, *CUC2* plays a major role in leaf serration development [41]. We find leaf serration is largely suppressed in the *cuc2 knat345* and _*pro*_ *35S*:*amiR* ^*159*^ *-KNAT345–1 cuc2* backgrounds (Fig. 5A-C and S14H-I Fig.). In addition, constitutive expression of KNOX2 (_*pro*_ *35S*:*KNAT3*) can partially suppress the _*pro*_ *BLS*:*STM* leaf phenotype (Fig. 5D-F). Thus, a common developmental program mediates both gain-of-function KNOX1 and loss-of-function KNOX2 leaf phenotypes.

As observed in _*pro*_ *BLS*:*STM* plants, elevated levels of KNOX1 activity are often associated with increased leaf complexity (reviewed in [11]). In *Cardamine hirsuta*, a close relative of *Arabidopsis*, dissected leaf development requires KNOX1 expression in leaves, and additional KNOX1 expression leads to ectopic leaflet initiation [42]. We investigated the outcome of reduction in the level of KNOX2 activity in this species. In *Cardamine*, leaf shape exhibits heteroblasty with leaflet number increasing in later produced leaves. Although leaflet number can vary for a particular leaf position, the first and second leaves always consist of a single, undivided, lamina, and the third leaf typically consisting of three leaflets (S15A Fig.). An amiRNA, *amiR* ^*159*^ *-KNAT345–2*, was designed to target three *Cardamine* genes homologous to *Arabidopsis KNAT3*, *KNAT4*, and *KNAT5*. Constitutive *amiR* ^*159*^ *-KNAT345–2* expression (_*pro*_ *35S*:*amiR* ^*159*^ *-KNAT345–2*; S7C Fig.) results in plants with an extra lateral leaflet on the second leaf, observed in approximately 15% of individuals (27 of 188 plants derived from 6 independent lines), indicating KNOX2 activity influences complexity of dissected leaves in *Cardamine* (Fig. 5G-H). Furthermore, gain-of-function KNOX2 alleles (_*pro*_ *35S*:*KNAT3*) simplify leaf shape, a phenotype particularly obvious in third leaves, which are undivided in strong lines (Fig. 5I-J and S15B Fig.). Thus, reduction or increase in KNOX2 activity leads to increase or decrease in leaf complexity, respectively, in *Cardamine* (Fig. 5 and S15 Fig.). This observation and the deduced KNOX1/KNOX2 antagonism are in consistent with the results in *Arabidopsis*.

## Discussion

*Arabidopsis* KNOX2 genes act redundantly to promote differentiation of all aerial organs in a manner broadly antagonistic to the action of KNOX1 genes. Loss-of-function KNOX2 alleles exhibit phenotypes with attributes of those of gain-of-function KNOX1 alleles, and vice versa, both in the maintenance of the shoot apical meristem and in the development of leaf complexity. In both contexts, KNOX2 functions to suppress meristematic capability, while KNOX1 promotes or maintains it. Our observations suggest that following the gene duplication giving rise to the KNOX1 and KNOX2 paralogs in an ancestor of land plants, neofunctionalization led to evolution of antagonistic biochemical activity thereby facilitating morphological evolution. Given the highly conserved nature of KNOX1 and KNOX2 genes in land plants, the antagonistic relationship may be a general phenomenon of diverse species.

Three *Arabidopsis* KNOX2 genes, *KNAT3*, *KNAT4*, and *KNAT5*, act redundantly in regulating plant development. Distinct phenotypes of double mutant combinations, however, indicate various degrees of contributions among three genes. For instance, *knat3 knat4* and *knat3 knat5* plants have more deeply serrated leaves, whereas *knat4 knat5* plants appear phenotypically wild type. Distinctive expression patterns may explain different phenotypic consequences in mutants [20], or alternatively, the potency of three KNOX2 proteins may vary owing to structural differences, and the different relative contributions of the three genes to leaf development can be seen as a process of subfunctionalization. Although *Arabidopsis* KNOX2 genes are expressed in the root, no overt phenotype was recognized in triple mutant roots, perhaps due to genetic redundancy with the fourth KNOX2 gene, *KNAT7*. In addition to their developmental roles, KNOX2 genes may play an undetected physiological role as they are expressed in senescing leaves (S4 Fig.) and have been reported to have a role involved in seed germination and early seedling development through modulating ABA responses [22]. Characterization of the quadruple mutants and physiological experiments may illuminate additional cryptic mutant phenotypes of KNOX2 genes.

### Evolution of leaf complexity

Expression of KNOX1 genes in leaves is correlated with increased leaf complexity and has been hypothesized to be influential in the evolution of leaf shape [42,43,44]. Given that seed plants leaves evolved from ancestral shoot systems, the ancestral seed plant leaf was likely complex, but fossil evidence and phylognetic analyses suggest that the ancestral angiosperm leaf may have been simple [45]. Regardless of the ancestral state, transitions from simple to more complex and vice versa have occurred repeatedly during angiosperm evolution [43,44,46]. In angiosperms, increase in leaf complexity is associated with increased KNOX1 activity while loss of KNOX1 activity in leaves results in decreasing complexity. While KNOX1 activity has been shown to play a pivotal role, other loci, such as *REDUCED COMPLEXITY* (*RCO*) in *Cardamine* and *LEAFY* (*LFY*) orthologues in legumes either contribute directly to modifying leaf shape or influence sensitivity to KNOX1 activity [11,47,48]. The lability of angiosperm leaf architecture may reflect that addition or loss of enhancer modules directing KNOX1 activity in leaves does not affect general plant viability.

The present study demonstrates that KNOX2 activities can also influence leaf shape—leaf dissection increases with decreasing KNOX2 activity (Fig. 2) in a dose dependent manner—raising the possibility of whether changes in KNOX2 activity could also have contributed to the evolution of leaf morphology. Just as KNOX1 gain-of-function alleles result in increases in leaf complexity, novel gain-of-function KNOX2 alleles that alter temporal or spatial expression patterns within developing leaves could contribute to the evolution from complex towards simple leaf morphology, as suggested by our experimental results in *Cardamine*, via acquisition of leaf specific enhancers. Alleles resulting in loss of KNOX2 activity could also contribute to increases in leaf complexity as suggested by the dose dependent changes to leaf shape in *Arabidopsis*, however, this may be less likely due to pleiotropic effects of loss-of-function KNOX2 alleles.

Intriguingly, in monilophytes KNOX1 gene expression is broadly similar to that of seed plants, with expression limited to less differentiated tissues including the shoot apical meristem, developing leaves, and procambial tissues [43,49,50]. KNOX2 gene expression has not been studied in detail, but similar to the situation in angiosperms, is reported to be throughout the sporophyte body [50]. In parallel with seed plants, simple leaves have evolved from more complex ancestral leaves within monilophytes [51]. Whether changes in KNOX1 or KNOX2 gene expression may be related to evolution of leaf form in monilophytes is presently unknown.

### Nature of the KNOX1/KNOX2 antagonistic relationship

One plausible explanation for the opposing action of KNOX1 and KNOX2 genes is an epistatic relationship between the gene classes. While non-overlapping expression patterns have been observed between KNOX1 and KNOX2 genes, we found no evidence for mutual repression. Alternatively, KNOX1 and KNOX2 proteins may interfere one another’s activity. Such a mode of action was proposed for KNATM in *Arabidopsis* and PETROSELINUM (PTS)/TKD1 in tomato, both of which are KNOX-related proteins that lack a DNA-binding homeodomain [52,53]. It is suggested that these mini KNOX proteins act as passive repressors and interfere with formation of a functional complex composed of canonical KNOX and BELL proteins. That KNOX2 function depends on the availability of appropriate BELL partners to be active, argues against a similar mechanism for the KNOX1/KNOX2 antagonism. Instead, our data favor a model whereby the antagonistic roles of KNOX1 and KNOX2 are at the level of opposing modes of transcriptional regulation.

Since addition of a repressor domain causes a dominant negative phenotype, KNOX1 proteins can act as activators [39,54]. Conversely a KNOX2 protein, KNAT7, can repress transcription in a transient protoplast system [18,19], and a motif similar to known repression domains is found in the ELK domain of all KNOX2 proteins [55] (S16 Fig.). Comparison of KNOX1 and KNOX2 homeodomains reveals that the third helices, an important determinant of DNA binding specificity, are highly conserved, indicating similar DNA binding properties, at least *in vitro* (S16 Fig.). Concurrently expressed KNOX1 and KNOX2 proteins could thus conceivably compete with each other at some target genes. Indeed, a putative KNOX2-SAW2 complex can overcome endogenous KNOX1 activities in the meristem, as does a dominant-negative form of KNOX1 (e.g., TKN2-SRDX [39] and en^298^-STM [54]). However, as KNOX1 proteins have also been reported to act to repress gene expression, the activity of KNOX proteins may be modified by either BELL partners, or third parties, such as OVATE proteins that interact with KNOX/BELL heterodimers and influence both their cellular localization and transcriptional activity [32,37,56]. In a related scenario, KNOX1 and KNOX2 could act on different sets of paralogs of downstream targets. These hypotheses are not mutually exclusive, and depending on the cellular contexts, different modes of action could operate, as is the case for the yeast TALE protein, Matα2, which has different partners in different cell types (reviewed in [23]).

Phylogenetic analyses indicate land plant KNOX1 and KNOX2 genes are derived from a single, ancestral KNOX gene. We hypothesize that subsequent to the KNOX1/KNOX2 gene duplication, accumulating structural differences endowed a new mode of action to at least one paralog. Therefore a possible evolutionary scenario could have an ancestral KNOX protein acting primarily as a transcriptional activator, with the evolution of a transcriptional repressor following gene duplication and neofunctionalization. The evolution of a repressor from an ancestral activator may be a common event, with several instances documented in plant transcription factor families [52,53,57,58,59,60]. Thus, within the context of land plant KNOX genes two types of negative regulators, in which the modes of repressor action are mechanistically different, may have evolved. Mini KNOX proteins act to inhibit KNOX activity by interacting with and sequestering BELL proteins [52,53], as opposed to antagonistic action at the level of downstream gene expression as we propose for KNOX2. The latter provides more flexibility due to the potential to act independently. Accompanying divergence in protein functionality, our data provides additional evidence for nearly complementary expression patterns of KNOX1 and KNOX2 genes in *Arabidopsis thaliana*. In contrast, in *P. patens* KNOX1 and KNOX2 genes exhibit both overlapping and distinctive expression patterns [14,26]. Changes in cis-regulatory sequences must have contributed to the establishment of complementary expression patterns during land plant evolution. Flexibility in gene regulatory networks governing meristematic maintenance and differentition engendered by the combination of changes in protein functionality and expression pattern could provide plasticity enabling morphological evolution.

### Diversification of KNOX/BELL modules

Heterodimerization between BELL and KNOX proteins is important for translocation of the complex into the nucleus [13]. BELL-KNOX2 heterodimerization may also be critical for providing specificity or increasing affinity of DNA binding (e.g. [61]). Although studies based on the yeast two-hybrid technique suggest physical interactions between BELL and KNOX proteins in a rather nonspecific manner [29,32], our genetic data suggest KNOX2 proteins interact *in planta* with a subset of BELL proteins, including those of the BEL1/SAW1/SAW2 clade. KNOX1 proteins rely on a distinct set of BELL proteins, e.g. PNY and PNF (reviewed in [12,13]). Due to an obligate heterodimerization requirement, the activity of a KNOX/BELL pair may be limited by the protein with the more restricted expression domain. In *Arabidopsis* KNOX2 functions appear to be regulated by restricted availability of corresponding BELL partners [29] (Fig. 3).

Similar to KNOX genes, land plant BELL genes evolved from a single gene in the algal ancestor [9]. However, the diversification of paralogs followed a different trajectory in the two families since BELL genes do not fall into discrete functional clades (S17 Fig.). For instance, KNOX1-interacting BELL genes (*PNY* and *PNF*) form a sister clade with KNOX2-interacting BELL genes (*BEL1* and *SAW1*/*2*). Moreover, genetic interactions implicate *BLH1*, from a phylogenetically distinct clade, as a KNOX2 partner since *knat3* alleles suppress the phenotype induced by ectopic *BLH1* embryo sac expression [37]. These phylogenetic relationships might be expected if the genome of the land plant common ancestor encoded a single BELL protein that interacted with both KNOX1 and KNOX2 proteins. As the BELL gene family diversified, subfunctionalization would have restricted interactions of BELL paralogs to specific KNOX1 or KNOX2 partners.

### KNOX1/KNOX2 gene duplication and land plant evolution

**Fig 6:**
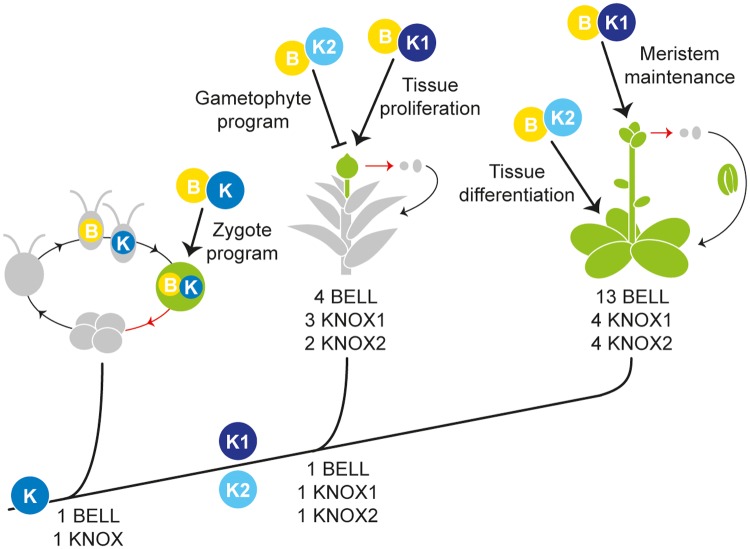
Proposed KNOX functions during land plant evolution. Along the phylogeny of plants, the primary functions for BELL (depicted as B) and KNOX (K) proteins, as well as the gene copy number, are presented for *Chlamydomonas* (a unicellular Chlorophyte alga), *Physcomitrella* (a moss), and *Arabidopsis* (a flowering plant). The ancestral conditions at branches were deduced from our phylogenetic analyses (S1 and S17 Figs.). In each life cycle, a red arrow indicates meiosis, and haploid (grey) and diploid (green) stages are color-coded. In *Chlamydomonas* the *plus* gamete expresses a BELL (depicted as B) protein while the *minus* gamete expresses a KNOX (K) protein; upon gamete fusion the KNOX and BELL proteins heterodimerize and regulate zygotic gene expression. Prior to the origin of land plants, a gene duplication in an ancestral KNOX gene generated two subclasses, KNOX1 (K1) and KNOX2 (K2) genes. In *Physcomitrella*, KNOX1 activity maintains tissue proliferation during sporophyte (diploid) development while KNOX2 represses the haploid genetic program during the diploid generation. In *Arabidopsis*, KNOX1 activity promotes meristem maintenance, and our study demonstrates that KNOX2 activity promotes tissue differentiation, perhaps via repression of meristematic functions, in the diploid generation. We propose that (1) the gene duplication producing KNOX1 and KNOX2 paralogs and ensuing neofunctionalization was instrumental in the evolution of a complex multicellular diploid generations in land plants and (2) the diversification of KNOX/BELL modules during land plant evolution facilitated the evolution of ever more complex diploid sporophyte body plans.

The defining feature of land plants is the formation of an embryo—a multicellular diploid generation. One prominent feature within land plant evolution is the transition from a gametophyte-dominant life cycle to a sporophyte-dominant life cycle [62,63]. This process is regarded as progressive sterilization and elaboration of vegetative organs [62], and in flowering plants, the gametophyte is reduced to a ephemeral structure of only a few cells that is dependent on a sporophyte body that can live up to thousands of years. If the ancestral KNOX-BELL genetic program regulated gene expression in a single celled zygote [25], it follows that during the course of land plant evolution, the KNOX/BELL module has been recruited to control numerous aspects of sporophyte development, with KNOX1/BELL modules promoting meristematic maintenance and continued growth and KNOX2/BELL modules promoting differentiation. In some cases, there is resemblance to a presumed ancestral function, such as in *P. patens* where KNOX2 genes regulate the gametophyte-to-sporophyte morphological transition [14,26]. In other cases, however, KNOX/BELL modules direct the development of novel structures, such as sporophyte shoot meristems and leaves (Fig. 6), that evolved later in land plant evolution, suggesting the duplication and diversification of the KNOX/BELL genetic module is linked with the evolution of morphological diversity in the land plant sporophyte. Neofunctionalization, exemplified by opposing activities between KNOX1 and KNOX2 genes in *Arabidopsis*, may underlie the molecular mechanism of key innovations and modification of body plans in the land plant history, through elaboration of transcriptional networks.

The role of TALE genes in fungi and *Chlamydomonas* can be viewed as promotion of cellular specialization in the diploid zygote and progression towards a meiotic state. The life cycle of land plants arose by an interpolation of mitotic divisions between fertilization and meiosis. Thus there is cell proliferation and a delay in meiosis in the diploid generation. KNOX1 genes prevent differentiation and maintain an undifferentiated state of the cells, enabling the cells to proliferate and develop a multicellular body in the sporophyte generation. In organisms with two heteromorphic multicellular generations, such as land plants, the developmental programs for each must be tightly controlled—a role suggested for KNOX2 genes in preventing the haploid gametophyte genetic program to be active during the diploid sporophyte generation in *Physcomitrella*. We hypothesize the duplication and diversification of the KNOX/BELL genetic module was instrumental in the evolution of a diploid embryo such that multicellular bodies develop in both haploid gametophyte and diploid sporophyte generations known as alternations of generations [25,26]. Alternations of generations have evolved independently in phylogenetically diverse eukaryotic lineages [64,65], prompting the question of whether similar TALE class genetic diversification may be found in these lineages.

## Materials and Methods

### Plant material and growth conditions

*Arabidopsis thaliana* accessions Columbia and Landsberg *erecta* (L*er*) were used as wild type in most experiments. _*pro*_ *KNAT2*:*GUS* was generated in the C24 background and introgressed into L*er. Cardamine hirsuta* ‘Oxford strain’ is a kind gift of A. Hay and M. Tsiantis. Plants were grown under long-day (18 hours light) or short-day (10 hours light) conditions at 20°C. *knat3* and *knat5* alleles are gift from V. Sundaresan and G. Pagnussat. *bp-9 knat2–5 knat6–1* seeds are gift from V. Pautot. T-DNA insertion alleles for BELL and KNOX genes were obtained from the Arabidopsis Biological Resource Center (ABRC) or the Nottingham Arabidopsis Stock Center (NASC). Mutant and transgenic lines have been described previously: *bp-9 knat2–5 knat6–1* [66]; *stm-11* [67]; _*pro*_ *BP*:*GUS* [68]; _*pro*_ *KNAT2*:*GUS* [69]; *Op*:*KNAT2* and *Op*:*STM* [39]; and _*pro*_ *STM*:*LhG4* [70]. The mutant and transgenic lines used in this study are listed in S1 Table. Homozygous mutant lines were identified by polymerase chain reaction (PCR)-based genotyping. Sequences of genotyping primers are available in S2 Table. The details of the transactivation system was previously described [33].

### Genetics

Multiple mutants combining *knat3*, *knat4*, and *knat5* alleles were generated by crossing, and genotypes were confirmed by PCR-based genotyping. To generate *bel1 blh1* double mutant, *blh1* plants were crossed with *bel1* plants, and the resulting F_2_ plants were examined. *bel1* plants were identified based on self-sterility, and among them, plants with yellow gynoecia segregated and were confirmed to be *bel1 blh1* double mutant plants by PCR-based genotyping. *knat2 knat3 knat5 knat6* and *bp knat345* plants were identified among F_2_ plants originating from a cross between *bp knat2 knat6* and *knat345* plants, and their genotypes were confirmed by PCR-based genotyping. *cuc2 knat345* plants were identified in a F_2_ population derived from a cross between *cuc2* and *knat345* plants. To generate _*pro*_ *FIL*:*miR164b* lines in the *knat345* mutant, self-fertile *knat3 knat5* plants were transformed with the _*pro*_ *FIL*:*miR164b* construct, and tranformants were selected by resistance to herbicide Basta. Single insertion lines were selected and crossed with *knat3/+ knat4 knat5* plants. Among F_1_ plants, self-fertile *knat3/+ knat4/+ knat5* plants carrying the _*pro*_ *FIL*:*miR164b* transgene were selected, and F_2_ seeds were collected; _*pro*_ *FIL*:*miR164b knat345* plants were identified in the resultant F_2_ population. To characterize the effects of the _*pro*_ *35S*:*amiR* ^*159*^ *-KNAT345–1* transgene in mutant backgrounds, the mutant plants were directly transformed with the _*pro*_ *35S*:*amiR* ^*159*^ *-KNAT345–1* construct, and transformants were selected by resistance to Basta. As *stm* null alleles are seedling lethal, heterozygous plants were used for transformation. More than twenty T_1_ plants for each background were examined, and phenotypes consistently observed among independent lines were reported.

### Semi-quantitative RT-PCR

RNA was extracted, using the RNeasy Plant Mini Kit (Qiagen), from 10-day-old seedlings grown on half-strength MS medium supplemented with 0.5% sucrose. RNA samples were treated with on-column DNaseI (Qiagen) and purified. SMARTScribe reverse transcriptase was used for cDNA synthesis (Clontech), and PCR reactions were performed using *Ex Taq* (Takara). Oligo sequences used for PCR reactions are described in S3 Table.

### Plasmid construction and plant transformation

amiRNAs were designed using the *Arabidopsis* pre-miR159a backbone (S7 Fig.) and synthesized (GenScript). For construction of the _*pro*_ *KNAT5*:*KNAT5-GUS* reporter construct, the genomic sequence spanning the *KNAT5* locus (from the next upstream annotated gene [At4g32030] to the next downstream annotated gene [At4g32050]) was used, and the stop codon was replaced with the GUS coding sequence. For construction of the _*pro*_ *KNAT4*:*GUS* reporter construct, an approximately 6.6-kb region of the sequence directly upstream of the *KNAT4* coding sequence was amplified using BAC T5K6 as PCR template and cloned into pCRII-TOPO (Invitrogen). The *KNAT4* upstream sequence was subcloned into the pRITA vector, which contains the GUS coding sequence and the terminator sequence from the nopaline synthase gene. For constitutive expression, the amiRNA sequences or the *KNAT3* coding sequence were cloned into the ART7 vector, which contains the Cauliflower mosaic virus _*pro*_ *35S* sequence and the terminator sequence from the octopine synthase gene. *KNAT5*, *SAW2*, and *PNY* coding sequences were amplified from L*er* cDNA and cloned downstream of an Lac Op array [33] to generate responder cassettes used in the transcription activation system. All constructs were subcloned into pMLBART or pART27 binary vector and were introduced into *Agrobacterium tumefaciens* strain GV3001 by electroporation. Transgenic lines were generated by *Agrobacterium*-mediated transformation, and transformants were selected on soil on the basis of resistance to the BASTA or kanamycin. Primers used to clone the various cDNAs and promoters are described in S2 Table.

### Histology and microscopy

Scanning electron microscopy was performed according to Alvarez and Smyth [71]. For light microscopy, cleared samples were prepared. Leaf samples were fixed overnight in 9:1 (v:v) ethanol:acetic acid at room temperature. After rehydration in a graded ethanol series, samples were rinsed with water and were cleared with chloral hydrate solution [1:8:2 (v:w:v) glycerol:chloral hydrate:water]. For histochemical analysis of GUS activity, samples were infiltrated with GUS staining solution [0.2% (w/v) Triton X-100, 2 mM potassium ferricyanide, 2 mM potassium ferrocyanide, and 1.9 mM 5-bromo-4-chloro-3-indolyl-β-glucuronide in 50 mM sodium phosphate buffer, pH 7.0] and incubated at 37°C.

### Phylogenetic analyses

Publically available KNOX and BELL coding nucleotide sequences representing taxa across land plants were manually aligned as amino acid translations using Se-Al v2.0a11 (http://tree.bio.ed.ac.uk/software/seal/). We excluded ambiguously aligned sequence to produce alignments for subsequent Bayesian analysis. Bayesian phylogenetic analysis was performed using Mr. Bayes 3.2.1 [72,73]. Three separate analyses were performed. The first included Chlorophyte algal and land plant KNOX sequences (S1 Fig.); the second included only land plant KNOX2 sequences (S2 Fig.); and the third included land plant BELL sequences (S17 Fig.). The fixed rate model option JTT + I was used based on analysis of the alignments with ProTest 2.4 [74]. Sequence alignments and command files used to run the Bayesian phylogenetic analyses are provided upon request.

## Supporting Information

S1 Fig Bayesian phylogram of KNOX genes. Numbers at branches indicate posterior probability values. Taxa are color coded according to major clades of taxa: magenta, algae; purple, moss; pale blue, lycophytes; dark blue, monilophyte; dark green, gymnosperms; pale green, angiosperms. *Arabidopsis* genes are marked with arrows. Class I (KNOX1) and class II (KNOX2) clades of land plant KNOX genes are indicated. Based on the tree topology, the common ancestor of mosses and flowering plants is predicted to have had a single KNOX1 gene and a single KNOX2 gene.
(TIF)

S2 Fig Bayesian phylogram of land plant KNOX2 genes. Numbers at branches indicate posterior probability values. Taxa are color coded according to major land plant clades: purple, moss; pale blue, lycophytes; dark blue, monilophyte; dark green, gymnosperms; pale green, angiosperms. Based on the tree topology, the gene duplication producing the KNAT7 and KNAT3/4/5 lineages occurred prior to the divergence between angiosperms and gymnosperms, about 300 Mya. The divergence may have occurred earlier, but additional sampling of fern and lycophyte lineages is required to clarify the timing.
(TIF)

S3 Fig Microarray expression data for *Arabidopsis* KNOX and BELL genes in meristematic cells. (**A-B**) KNOX and BELL expression in inflorescence meristem cells expressing fluorescent reporters, _*pro*_ *CLV3*:*mGFP5-ER* (**A**) or _*pro*_ *WUS*:*mGFP5-ER* (**B**). *CLV3* (AT2G27250, expressed in the shoot apical meristem), *WUS* (AT2G17950, expressed in the shoot apical meristem), *PHB* (AT2G34710, expressed in the shoot apical meristem and in the adaxial side of lateral organs), and *FIL* (AT2G45190, expressed in the abaxial side of lateral organs) expression levels are shown as references. KNOX1 genes and a subset of BELL genes (*BLH7*, *PNF*, and *PNY*) are expressed in meristematic cell types whereas KNOX2 and their BELL partners (*BEL1*, *SAW1*, and *SAW2*) are expressed at low levels or are not detected in meristematic cells. Key: KNOX1 genes and presumptive KNOX1-interacting BELL genes are color-coded in green. KNOX2 genes and presumptive KNOX2-interacting BELL genes are color-coded in purple. BELL-KNOX genetic interactions are described in previous [22,37,75,76,77] and present studies. Expression patterns of *BLH3* and *BLH10* are tightly linked to those of KNOX2 genes, indicating potential interactions [78]. Error bars denote standard deviations. Microarray data by cell-type specific expression analysis using cells derived from the inflorescence meristem [79] was retrieved through Arabidopsis eFP Browser (http://bar.utoronto.ca/efp/cgi-bin/efpWeb.cgi; [80]).
(TIF)

S4 Fig Microarray expression data for *Arabidopsis* KNOX and BELL genes in differentiating leaves. (**A-B**) KNOX and BELL expression in young (**A**) and senescing (**B**) leaves of wild-type plants. KNOX2 genes are abundantly expressed in these tissues whereas KNOX1 expression is low or not detectable. Key: Reference genes and color codes are as per S3 Fig. Error bars denote standard deviations. Microarray data were retrieved through Arabidopsis eFP Browser (http://bar.utoronto.ca/efp/cgi-bin/efpWeb.cgi; [80]). Sample descriptions and identifiers are as follows: (**A**) first and second leaves from 7-day-old plants from the ATGE_5 dataset; (**B**) senescing leaves from 35-day-old plants from the ATGE_25 dataset.
(TIF)

S5 Fig Expression of KNOX2 genes in mutant backgrounds. RNA was isolated from 10-day-old wild-type Columbia (designated as wt), *knat4* (4), and *knat345* (tri) plants, and expression levels of *KNAT3*, *KNAT4*, and *KNAT5* genes were analyzed by semi-quantitative RT-PCR. Cyclophilin (AT2G29960) expression was examined as internal control. Genomic DNA (g) isolated from wild-type Columbia plants was included for analysis.
(TIF)

S6 Fig Venation patterning defects in cotyledons of KNOX2 mutants. (**A-C**) Venation patterns of wild-type (**A**), *knat3 knat5* (**B**), and *knat345* (**C**) cotyledons. Discontinuous venation is observed in the distal part of *knat3 knat5* cotyledons. In *knat345* cotyledons, the venation pattern is simplified and consists of a single primary vein. (**D-F**) The distal parts of wild-type (**D**), *knat3 knat5* (**E**), and *knat345* (**F**) cotyledons at higher magnification to show vascular strands. Consistent with the mutant phenotype, _*pro*_ *KNAT5*:*KNAT5-GUS* expression was detected along cotyledon veins (see Fig. 2T). Plants are in the Col background and grown for 1 week. Scale bars in **A-C**, 500 μm and in **D-F**, 100 μm.
(TIF)

S7 Fig Design of amiRNAs used in this study. (**A**) Design of the *amiR* ^*159*^ *-KNAT4*, which specifically targets *Arabidopsis KNAT4* gene, embedded in pre-miR159a fold-back structure. (**B**) Design of the *amiR* ^*159*^ *-KNAT345–1*, which targets *KNAT3*, *KNAT4*, and *KNAT5* genes in *Arabidopsis*, embedded in pre-miR159a fold-back structure. (**C**) Design of the *amiR* ^*159*^ *-KNAT345–2* embedded in pre-miR159a fold-back structure. The *amiR* ^*159*^ *-KNAT345–2* was designed to target *KNAT3*, *KNAT4*, and *KNAT5* genes in *Arabidopsis thaliana* as well as *Cardamine hirsuta* orthologues to these genes, *ChKN3*, *ChKN4*, and *ChKN5* (M. Tsiantis, personal communication). The predicted fold-back structures are presented with amiRNA sequences highlighted in red. The mfold web server (http://mfold.rna.albany.edu/?q=mfold/RNA-Folding-Form; [81]) was used to predict secondary structures.
(TIF)

S8 Fig Leaf phenotype of _*pro*_ *35S:amiR* ^*159*^ *-KNAT4 knat3 knat5* and _*pro*_ *35S:amiR* ^*159*^ *-KNAT345–1* plants. (**A-E**) Whole plant images of wild-type (**A**), *knat3 knat5* (**B**), *knat345* (**C**), *knat3 knat5* plants expressing _*pro*_ *35S*:*amiR* ^*159*^ *-KNAT4* (**D**), and _*pro*_ *35S*:*amiR* ^*159*^ *-KNAT345–1* (**E**) plants. Constitutive expression of *amiR* ^*159*^ *-KNAT4* in *knat3 knat5* (**D**) and constitutive expression of *amiR* ^*159*^ *-KNAT345–1* (**E**) recapitulate the leaf serration phenotype of *knat345* plants. Plants are in the Col background. Plants in (**A, B, D**) are 5 weeks old, and plants in (**C, E**) are one month old.
(TIF)

S9 Fig Gynoecium development in KNOX2 loss-of-function and *bel1* mutants. (**A-D**) An inflorescence apex and a series of developing flowers, pistils or fruits detached from it are arranged from left to right. (**A**) Wild type. (**B**) *knat3 knat4*. (**C**) *knat345*. (**D**) *bel1–154*. Some wild-type flowers were emasculated and left unpollinated (indicated as UP). In *knat3 knat4* (**B**) and *knat345* (**C**) plants, the color of the valve and the replum turns into yellow. This is independent from female sterility of *knat345* plants since the color of the unpollinated gynoecium stays green in wild-type plants (**A**). Note that the yellowing phenotype is stronger in *knat345* than in *knat3 knat4*. The gynoecia of *bel1–154* single mutant plants do not show change in color. Plants are in the Col background. Scale bars, 1 mm.
(TIF)

S10 Fig The morphology and anatomy of *knat345* roots. (**A-B**) 5-day-old wild-type (**A**) and *knat345* (**B**) seedlings grown on nutrient agar plates. (**C-D**) DIC (differential interference contrast) optical sections through the root meristems of wild-type (**C**) and *knat345* (**D**) plants. Plants are in the Col background. Scale bars in **A, B**, 1 mm and in **C, D**, 50 μm.
(TIF)

S11 Fig _*pro*_ *KNAT5:KNAT5-GUS* expression patterns. (**A, B**) _*pro*_ *KNAT5*:*KNAT5-GUS* expression was detected in developing leaves. Reduced signal levels were observed in older leaves (**B**). (**C**) _*pro*_ *KNAT5*:*KNAT5-GUS* activity is excluded from the shoot apical meristem (marked by an arrowhead). No detectable _*pro*_ *KNAT5*:*KNAT5-GUS* signal was observed in the stipule (marked with an asterisk). (**D-G**) A series of leaves showing _*pro*_ *KNAT5*:*KNAT5-GUS* expression from early (**D**) to late (**G**) stages of leaf development. Expression was first detected throughout leaf (**D**) and later becomes more restricted towards the proximal part of the lamina with strong expression in developing teeth (**F-G**). _*pro*_ *KNAT5*:*KNAT5-GUS* expression was also detected in some stipules (marked with asterisks). (**H-J**) Prolonged, overnight incubation detected _*pro*_ *KNAT5*:*KNAT5-GUS* along the vascular system in leaves (**H-I**) and gynoecia of flowers (**J**). (**A-B, F, I**) _*pro*_ *KNAT5*:*KNAT5-GUS* expression was detected in the nucleus of trichomes. Plants are in the Col background. Scale bars in **C-E**, 50 μm and in **I**, 500 μm.
(TIF)

S12 Fig Morphology of _*pro*_ *35S:KNAT3* plants. (**A-D**) Morphology of wild-type (**A, C**) and homozygous _*pro*_ *35S*:*KNAT3* (**B, D**) plants. (**A, B**) Compared to wild-type (**A**), the shape of _*pro*_ *35S*:*KNAT3* plants is more compact with shorter petioles and slightly smaller leaves (**B**). (**C-D**) The stems of _*pro*_ *35S*:*KNAT3* plants (**D**) are shorter than those of wild-type (**C**). (**E-F**) Wild-type (**E**) and _*pro*_ *35S*:*KNAT3* (**F**) flowers, showing fusion between sepals in _*pro*_ *35S*:*KNAT3* flowers. Plants are in the Col background. Plants in (A, B), (C), and (D) are 12 days old, 6 weeks old, and 5 weeks old, respectively.
(TIF)

S13 Fig Floral morphology in _*pro*_ *STM>>BELL* and _*pro*_ *STM>>KNOX* plants. (**A, G**) Wild-type L*er* inflorescences. (**B-F, H**) Inflorescences of transactivation lines expressing *SAW2* (**B, H**), *PNY* (**C**), *STM* (**D**), *KNAT2* (**E**), or *KNAT5* (**F**) under the control of the *STM* regulatory sequence, _*pro*_ *STM*. Expression of KNOX1 (e.g., *STM* and *KNAT2*), KNOX2 (e.g., *KNAT5*), or *PNY* does not impact flower development whereas abnormal phenotypes, such as fused sepals, reduced petals, and misshapen fruits, are observed in _*pro*_ *STM>>SAW2* flowers (**B, H**). Flower development was not impacted in _*pro*_ *STM>>KNAT5* plants, but fused sepals are observed in strong _*pro*_ *35S*:*KNAT3* lines (S12F Fig.). Although studies based on the yeast two-hybrid technique suggest physical interactions between BELL and KNOX proteins in a rather nonspecific manner [29,32], the genetic data here and in Fig. 3 suggest KNOX2 proteins interact *in planta* with a subset of BELL proteins, including those of the BEL1/SAW1/SAW2 clade. KNOX1 proteins rely on a distinct set of BELL proteins, e.g. PNY and PNF [75,76]. Due to an obligate heterodimerization requirement, the activity of a KNOX/BELL pair may be limited by the protein with the more restricted expression domain. In *Arabidopsis*, KNOX2 functions appear to be regulated by restricted availability of corresponding BELL partners. Plants are in the L*er* background.
(TIF)

S14 Fig The *cuc2* genetic background suppresses the leaf lobing of ectopic KNOX1 and reduced KNOX2 activities. (**A-B**) Wild-type (**A**) and a plant with *STM* expression driven by the leaf specific promoter, _*pro*_ *BLS*, _*pro*_ *BLS*:*STM* (**B**), exhibiting a deeply lobed leaf phenotype characteristic of gain-of-function KNOX1 alleles. Plants are 6 weeks old. (**C**) From left to right are _*pro*_ *BLS>>miR164b*, _*pro*_ *BLS>>STM*, and _*pro*_ *BLS>>STM/miR164b* plants, where either *miR164b* or *STM* alone, or both genes together, are transactivated by _*pro*_ *BLS*. The miR164 family of miRNAs target CUC genes including *CUC2*, a key regulator of leaf serration. Leaf-specific miR164 expression largely suppresses serration development along the lamina margin both in wild-type and _*pro*_ *BLS>>STM* backgrounds. Plants are grown at the same time under the short-day conditions. (**D-E**) Wild-type (**D**) and _*pro*_ *FIL*:*miR164b* (**E**), where *miR164b* expression is driven in young leaves using regulatory sequences of the *FILAMENTOUS FLOWER* (*FIL*) gene (designated as _*pro*_ *FIL*), plants. As in _*pro*_ *BLS>>miR164b* plants, leaf serration is largely suppressed in _*pro*_ *FIL*:*miR164b* plants. (**F-G**) _*pro*_ *FIL*:*miR164b* suppresses the leaf serration phenotype of *knat345* plants. Plants shown in (**D-G**) were grown for 25 days under short-day conditions. (**H-I**) Similarly, the *cuc2* mutation largely suppresses the leaf serration phenotype of _*pro*_ *35S*: *amiR* ^*159*^ *-KNAT345–1* plants. Close-ups of 7 weeks-old plants are shown. Plants in (C) are in the L*er* background, and other plants are in the Col background. Scale bars in **A, B**, 1 cm.
(TIF)

S15 Fig Constitutive KNOX2 expression in *Cardamine hirsuta*. (**A-B**) Wild-type (**A**) and _*pro*_ *35S*:*KNAT3* (**B**) leaves, removed from single plants and arranged in acropetal sequence (oldest to youngest) from left to right. In *Cardamine hirsuta*, leaf shape exhibits heteroblasty with leaflet number increasing in later produced leaves. Although leaflet number can vary for a particular leaf position, the first and second leaves always consist of a single, undivided, lamina, and the third leaf typically consists of three leaflets (marked by an arrow in **A**). (**B**) Gain-of-function KNOX2 allele (constitutive expression of the *KNAT3* gene from *Arabidopsis*; _*pro*_ *35S*:*KNAT3*) in *Cardamine hirsuta* simplifies leaf shape, a phenotype particularly obvious in third leaves (indicated by arrows), which are undivided in strong lines. Plants are grown for one month. Scale bars, 1 mm.
(TIF)

S16 Fig Alignment of the deduced amino acid sequences of algal and land plant KNOX proteins. Amino acids identical to the one at the equivalent position in the *Arabidopsis* KNAT3 sequence are indicated with dots. Dashes denote a lack of corresponding sequence from the *Arabidopsis* KNAT3 sequence. Amino acid sequences that form three helices in the homeodomain are indicated. KNOX2, KNOX1, and algal KNOX sequences are color coded in magenta, green, and blue, respectively. The region encompassing the position of a presumptive KNOX2 repression motif (highlighted in yellow) and the homeodomain (highlighted in pale blue) is presented. The putative repression motif is absent in land plant KNOX1 and algal KNOX proteins and is one of the structural differences between KNOX1 and KNOX2 proteins [9]. Comparison of KNOX1 and KNOX2 homeodomains reveals that the third helices, an important determinant of DNA binding specificity, are highly conserved, indicating similar DNA binding properties, at least *in vitro*.
(TIF)

S17 Fig Bayesian phylogram of land plant BELL genes. Numbers at branches indicate posterior probability values. Taxa are color coded according to major land plant clades: purple, moss; blue, lycophyte; dark green, gymnosperms; pale green, angiosperms. Clades that include *Arabidopsis* genes with known functions are indicated. *Arabidopsis* genes are highlighted using a larger font. Three *Arabidopsis* genes, *ATH1*, *BLH5*, and *BLH11*, were not included in this analysis because the sequences are divergent from those of other genes and cause long branch attraction and tree distortion. The approximate phylogenetic positions of these genes are indicated next to the phylogram with their names presented in parentheses. Based on genetic evidence (genetics) or overlapping expression patterns (exp. patterns) obtained from the previous and current studies, presumptive heterodimeric partners for *Arabidopsis* BELL proteins are postulated and placed beside the clades: K1 and K2 denoting KNOX1 and KNOX2 proteins, respectively. Ambiguous interactions are indicated by question marks. References for BELL-KNOX interactions are as per S3 Fig. Expression patterns were analyzed using ATTED-II (http://atted.jp/; [78]). Similar to KNOX genes, land plant BELL genes evolved from a single gene in the algal ancestor [8]. Note that the diversification of paralogs, however, followed a different trajectory in the two families as BELL genes do not fall into discrete functional clades. Namely, KNOX1-interacting BELL genes (*PNY* and *PNF*) form a sister clade with KNOX2-interacting BELL genes (*BEL1* and *SAW1*/*2*), while genetic interactions implicate *BLH1*, from a phylogenetically distinct clade, as a KNOX2 partner since *knat3* alleles suppress the phenotype induced by ectopic *BLH1* expression [22,37].
(TIF)

S1 Table Mutant and transgenic lines used in this study. (DOCX)

S2 Table Primers used in this study. (DOCX)

S3 Table Primers used for semi-quantitative RT-PCR. (DOCX)
