# Supplementary figures and images for: Antagonistic Roles for KNOX1 and KNOX2 Genes in Patterning the Land Plant Body Plan Following an Ancient Gene Duplication
Source: PLoS Genet. 2015 Feb 11;11(2):e1004980. doi: 10.1371/journal.pgen.1004980 (PMC4335488; doi:10.1371/journal.pgen.1004980)

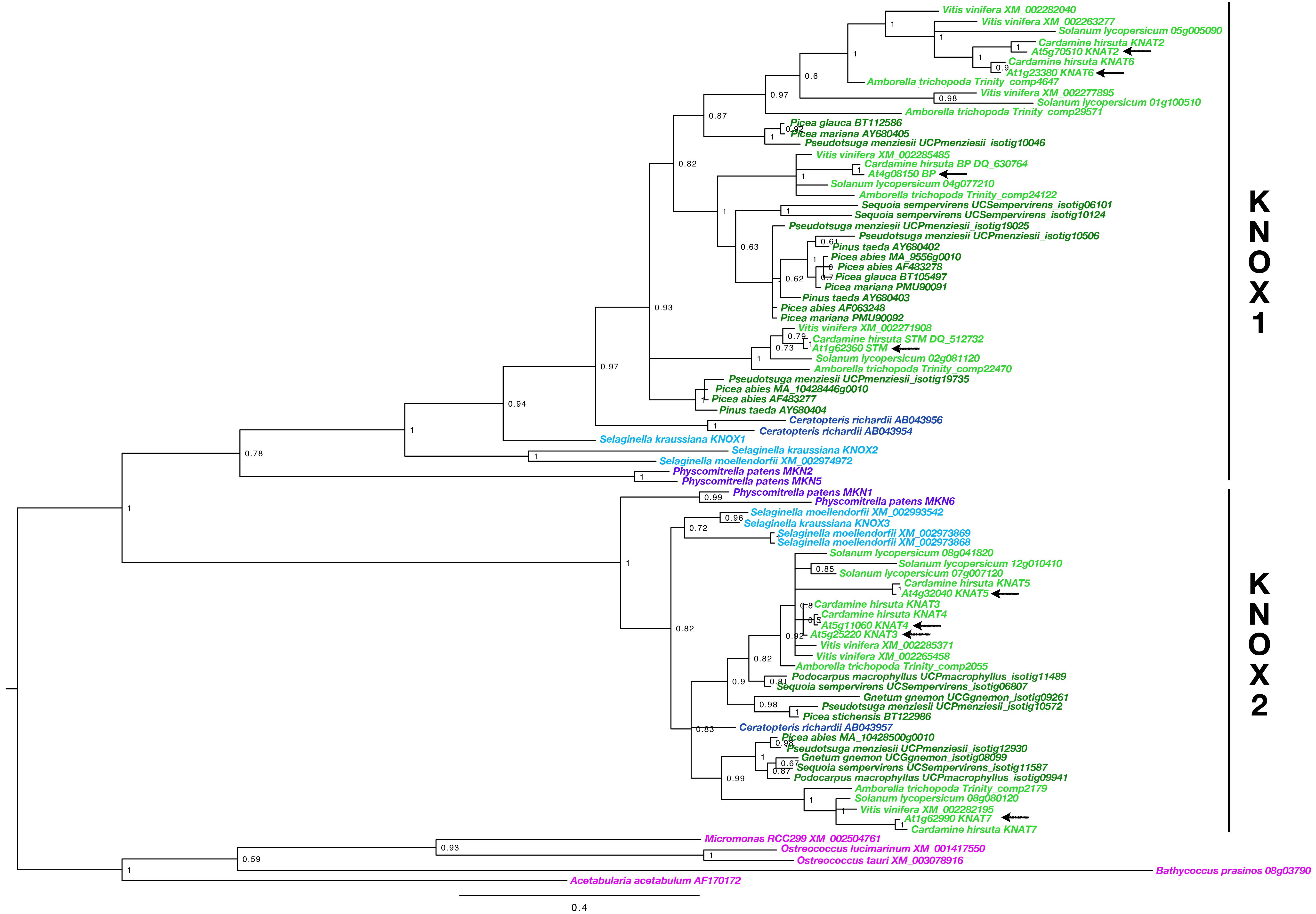

Supplement: S1 Fig — Numbers at branches indicate posterior probability values. Taxa are color coded according to major clades of taxa: magenta, algae; purple, moss; pale blue, lycophytes; dark blue, monilophyte; dark green, gymnosperms; pale green, angiosperms. Arabidopsis genes are marked with arrows. Class I (KNOX1) and class II (KNOX2) clades of land plant KNOX genes are indicated. Based on the tree topology, the common ancestor of mosses and flowering plants is predicted to have had a single KNOX1 gene and a single KNOX2 gene. (TIF) [file pgen.1004980.s001.tif]

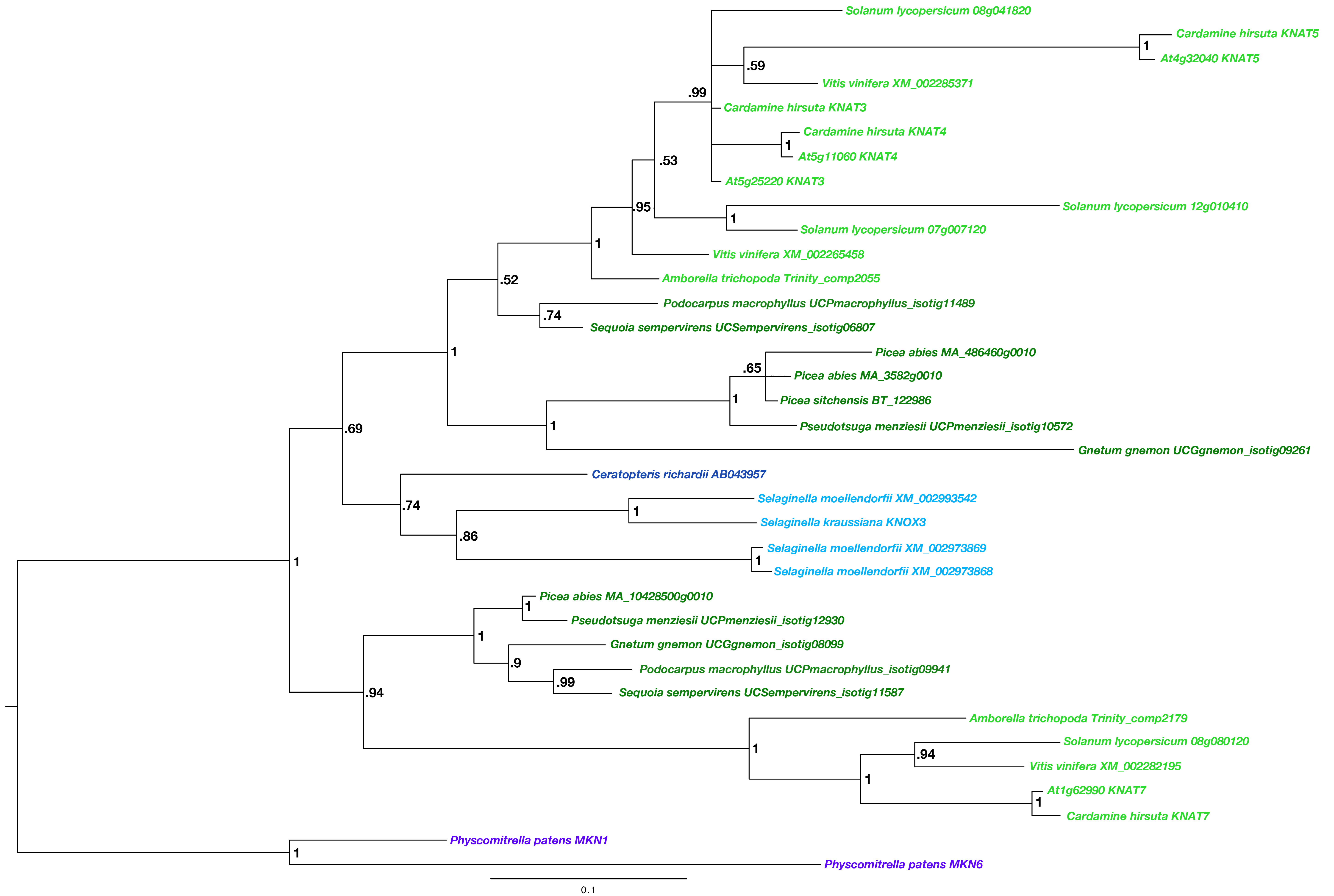

Supplement: S2 Fig — Numbers at branches indicate posterior probability values. Taxa are color coded according to major land plant clades: purple, moss; pale blue, lycophytes; dark blue, monilophyte; dark green, gymnosperms; pale green, angiosperms. Based on the tree topology, the gene duplication producing the KNAT7 and KNAT3/4/5 lineages occurred prior to the divergence between angiosperms and gymnosperms, about 300 Mya. The divergence may have occurred earlier, but additional sampling of fern and lycophyte lineages is required to clarify the timing. (TIF) [file pgen.1004980.s002.tif]

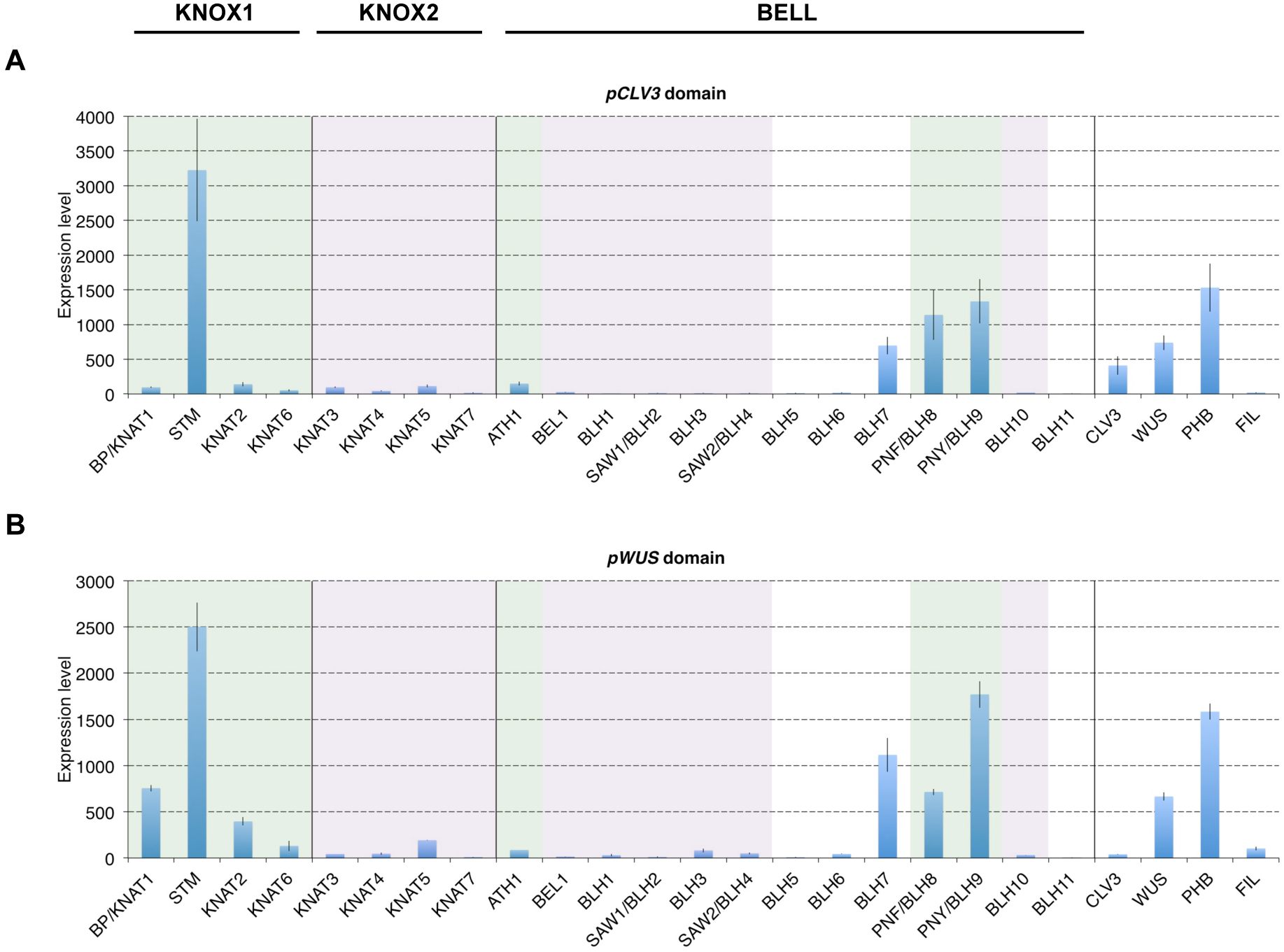

Supplement: S3 Fig — (A-B) KNOX and BELL expression in inflorescence meristem cells expressing fluorescent reporters, pro CLV3:mGFP5-ER (A) or pro WUS:mGFP5-ER (B). CLV3 (AT2G27250, expressed in the shoot apical meristem), WUS (AT2G17950, expressed in the shoot apical meristem), PHB (AT2G34710, expressed in the shoot apical meristem and in the adaxial side of lateral organs), and FIL (AT2G45190, expressed in the abaxial side of lateral organs) expression levels are shown as references. KNOX1 genes and a subset of BELL genes (BLH7, PNF, and PNY) are expressed in meristematic cell types whereas KNOX2 and their BELL partners (BEL1, SAW1, and SAW2) are expressed at low levels or are not detected in meristematic cells. Key: KNOX1 genes and presumptive KNOX1-interacting BELL genes are color-coded in green. KNOX2 genes and presumptive KNOX2-interacting BELL genes are color-coded in purple. BELL-KNOX genetic interactions are described in previous [22,37,75,76,77] and present studies. Expression patterns of BLH3 and BLH10 are tightly linked to those of KNOX2 genes, indicating potential interactions [78]. Error bars denote standard deviations. Microarray data by cell-type specific expression analysis using cells derived from the inflorescence meristem [79] was retrieved through Arabidopsis eFP Browser (http://bar.utoronto.ca/efp/cgi-bin/efpWeb.cgi; [80]). (TIF) [file pgen.1004980.s003.tif]

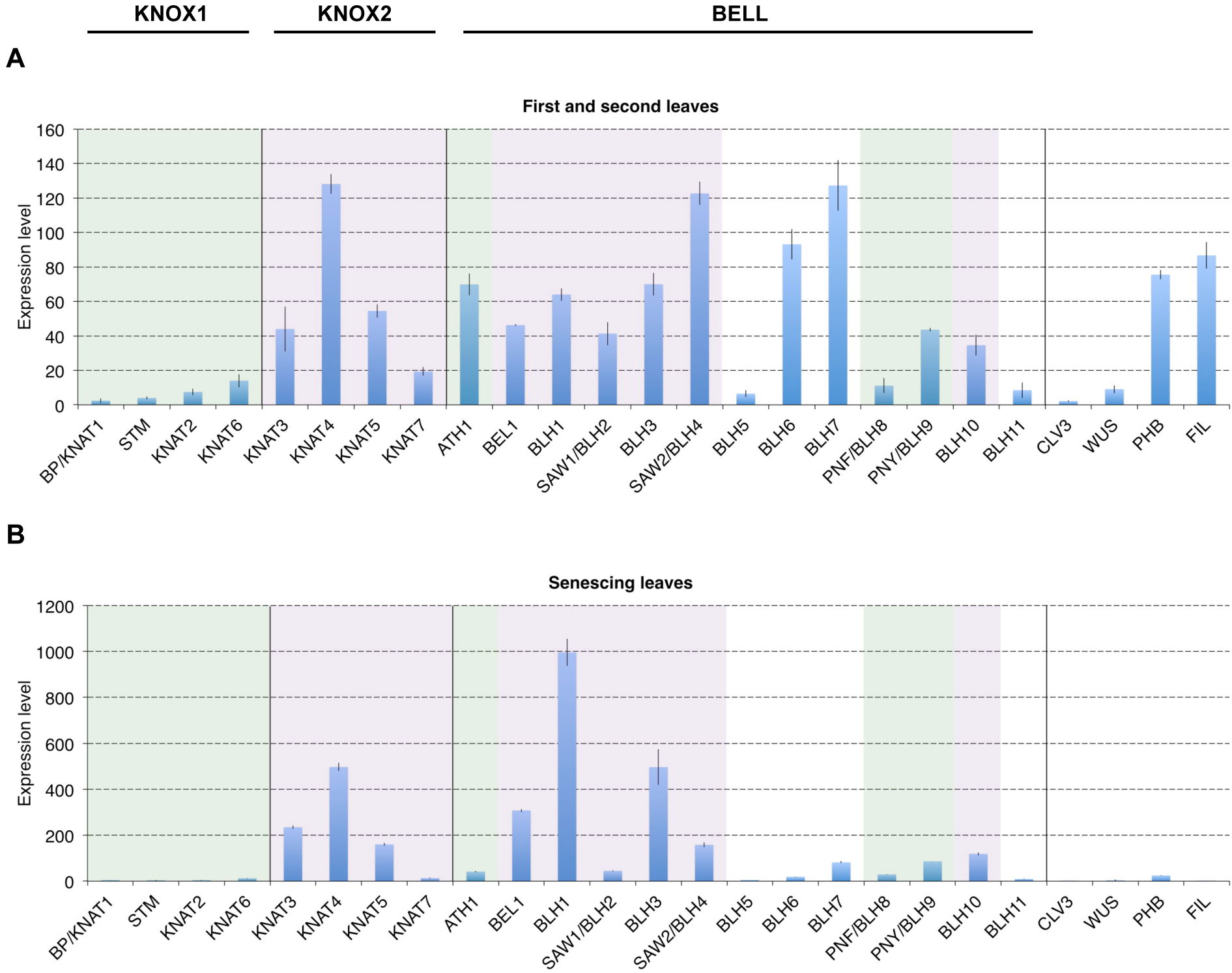

Supplement: S4 Fig — (A-B) KNOX and BELL expression in young (A) and senescing (B) leaves of wild-type plants. KNOX2 genes are abundantly expressed in these tissues whereas KNOX1 expression is low or not detectable. Key: Reference genes and color codes are as per S3 Fig. Error bars denote standard deviations. Microarray data were retrieved through Arabidopsis eFP Browser (http://bar.utoronto.ca/efp/cgi-bin/efpWeb.cgi; [80]). Sample descriptions and identifiers are as follows: (A) first and second leaves from 7-day-old plants from the ATGE_5 dataset; (B) senescing leaves from 35-day-old plants from the ATGE_25 dataset. (TIF) [file pgen.1004980.s004.tif]

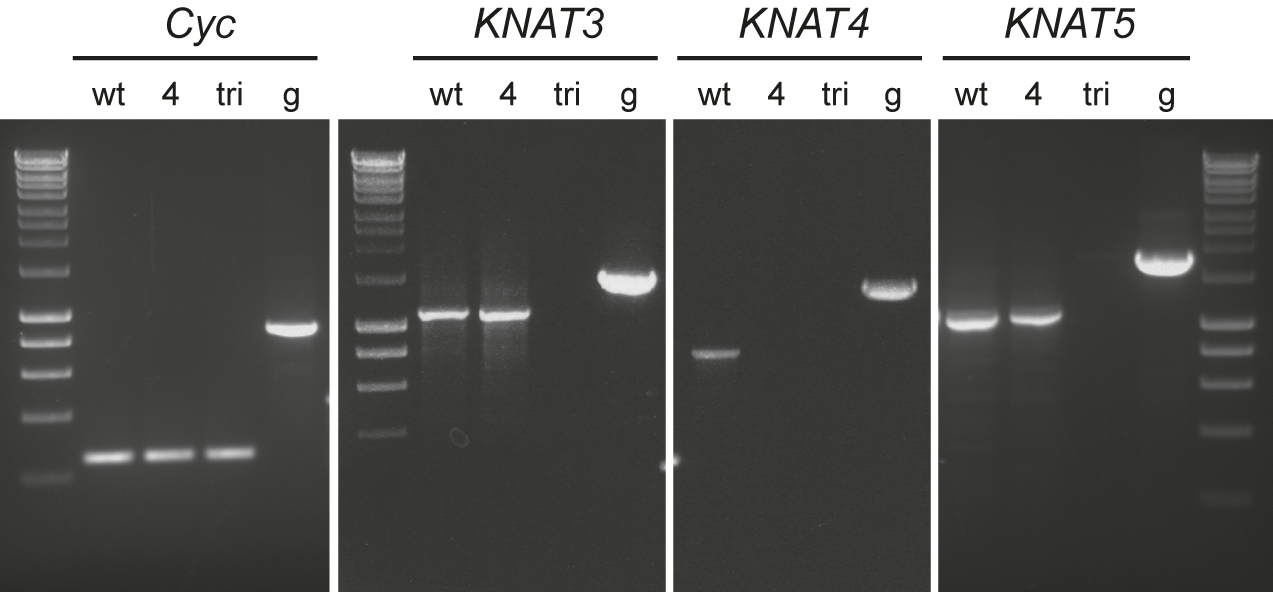

Supplement: S5 Fig — RNA was isolated from 10-day-old wild-type Columbia (designated as wt), knat4 (4), and knat345 (tri) plants, and expression levels of KNAT3, KNAT4, and KNAT5 genes were analyzed by semi-quantitative RT-PCR. Cyclophilin (AT2G29960) expression was examined as internal control. Genomic DNA (g) isolated from wild-type Columbia plants was included for analysis. (TIF) [file pgen.1004980.s005.tif]

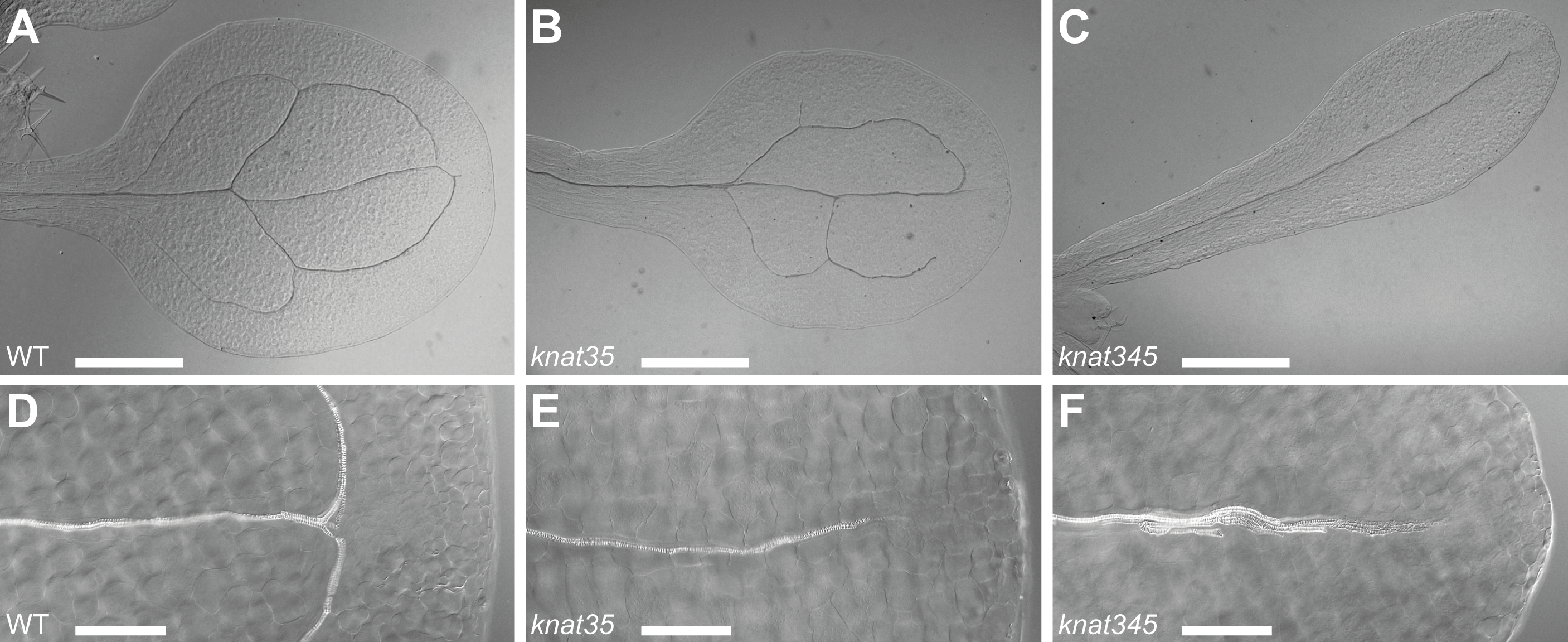

Supplement: S6 Fig — (A-C) Venation patterns of wild-type (A), knat3 knat5 (B), and knat345 (C) cotyledons. Discontinuous venation is observed in the distal part of knat3 knat5 cotyledons. In knat345 cotyledons, the venation pattern is simplified and consists of a single primary vein. (D-F) The distal parts of wild-type (D), knat3 knat5 (E), and knat345 (F) cotyledons at higher magnification to show vascular strands. Consistent with the mutant phenotype, pro KNAT5:KNAT5-GUS expression was detected along cotyledon veins (see Fig. 2T). Plants are in the Col background and grown for 1 week. Scale bars in A-C, 500 μm and in D-F, 100 μm. (TIF) [file pgen.1004980.s006.tif]

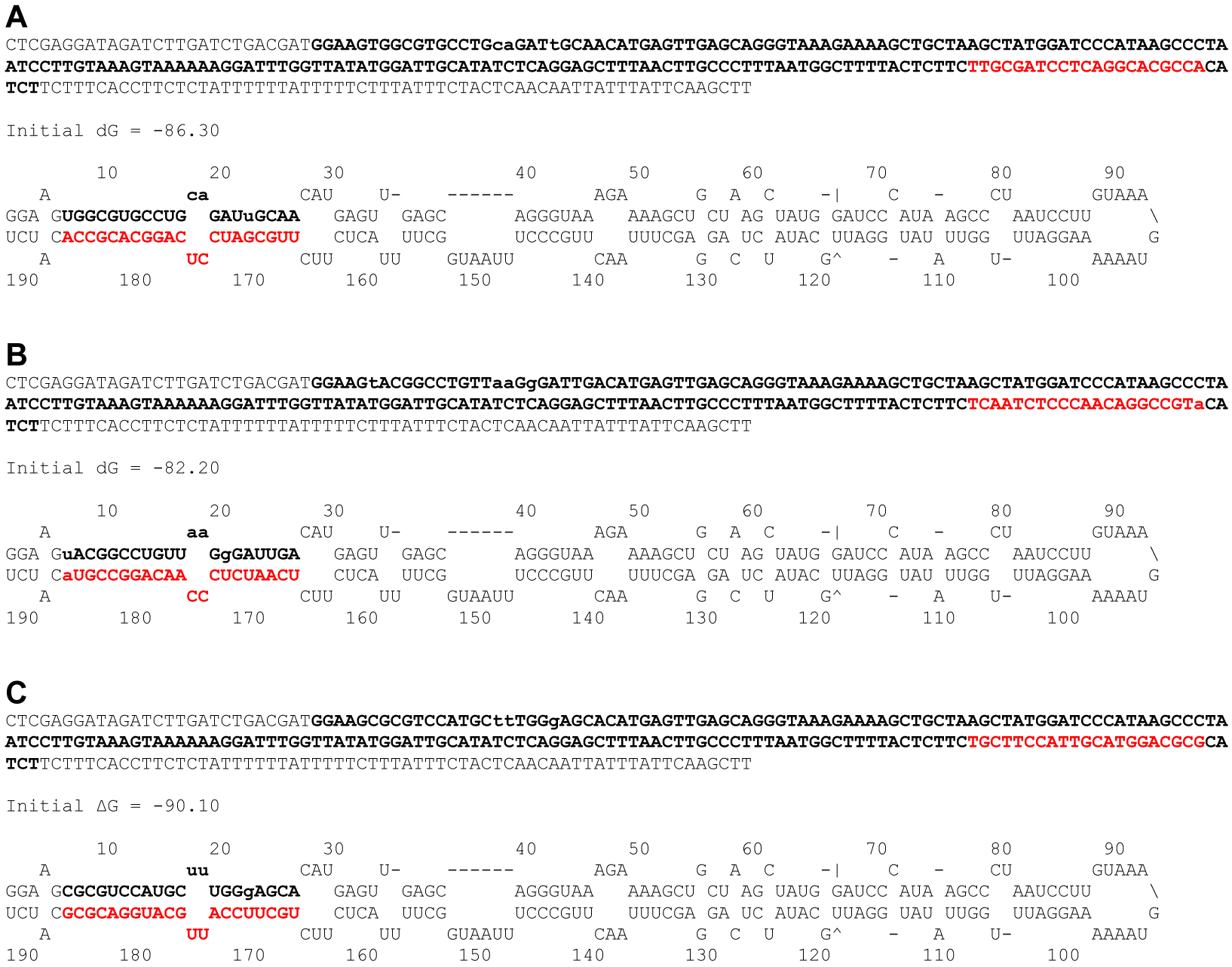

Supplement: S7 Fig — (A) Design of the amiR 159 -KNAT4, which specifically targets Arabidopsis KNAT4 gene, embedded in pre-miR159a fold-back structure. (B) Design of the amiR 159 -KNAT345–1, which targets KNAT3, KNAT4, and KNAT5 genes in Arabidopsis, embedded in pre-miR159a fold-back structure. (C) Design of the amiR 159 -KNAT345–2 embedded in pre-miR159a fold-back structure. The amiR 159 -KNAT345–2 was designed to target KNAT3, KNAT4, and KNAT5 genes in Arabidopsis thaliana as well as Cardamine hirsuta orthologues to these genes, ChKN3, ChKN4, and ChKN5 (M. Tsiantis, personal communication). The predicted fold-back structures are presented with amiRNA sequences highlighted in red. The mfold web server (http://mfold.rna.albany.edu/?q=mfold/RNA-Folding-Form; [81]) was used to predict secondary structures. (TIF) [file pgen.1004980.s007.tif]

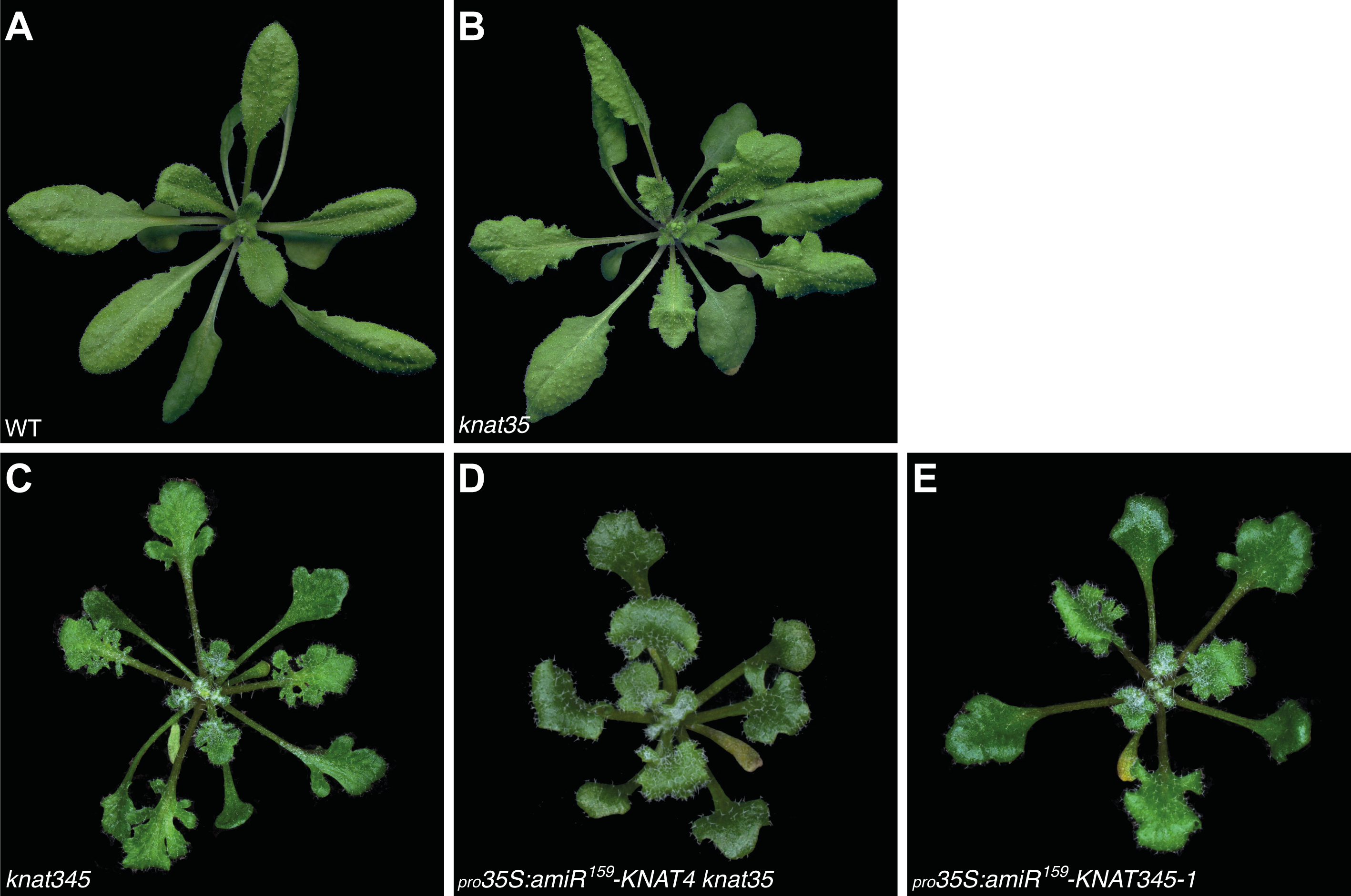

Supplement: S8 Fig — (A-E) Whole plant images of wild-type (A), knat3 knat5 (B), knat345 (C), knat3 knat5 plants expressing pro 35S:amiR 159 -KNAT4 (D), and pro 35S:amiR 159 -KNAT345–1 (E) plants. Constitutive expression of amiR 159 -KNAT4 in knat3 knat5 (D) and constitutive expression of amiR 159 -KNAT345–1 (E) recapitulate the leaf serration phenotype of knat345 plants. Plants are in the Col background. Plants in (A, B, D) are 5 weeks old, and plants in (C, E) are one month old. (TIF) [file pgen.1004980.s008.tif]

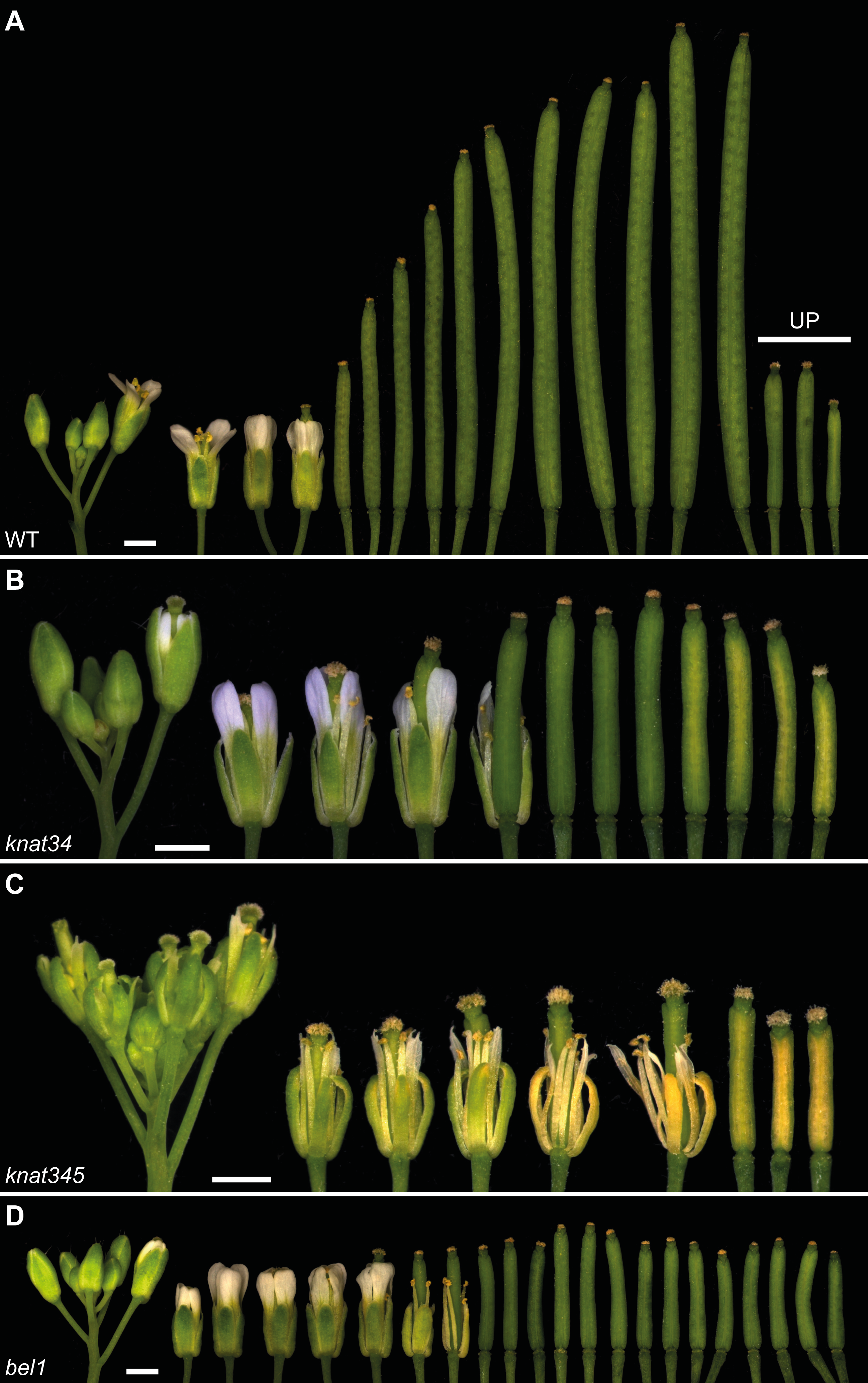

Supplement: S9 Fig — (A-D) An inflorescence apex and a series of developing flowers, pistils or fruits detached from it are arranged from left to right. (A) Wild type. (B) knat3 knat4. (C) knat345. (D) bel1–154. Some wild-type flowers were emasculated and left unpollinated (indicated as UP). In knat3 knat4 (B) and knat345 (C) plants, the color of the valve and the replum turns into yellow. This is independent from female sterility of knat345 plants since the color of the unpollinated gynoecium stays green in wild-type plants (A). Note that the yellowing phenotype is stronger in knat345 than in knat3 knat4. The gynoecia of bel1–154 single mutant plants do not show change in color. Plants are in the Col background. Scale bars, 1 mm. (TIF) [file pgen.1004980.s009.tif]

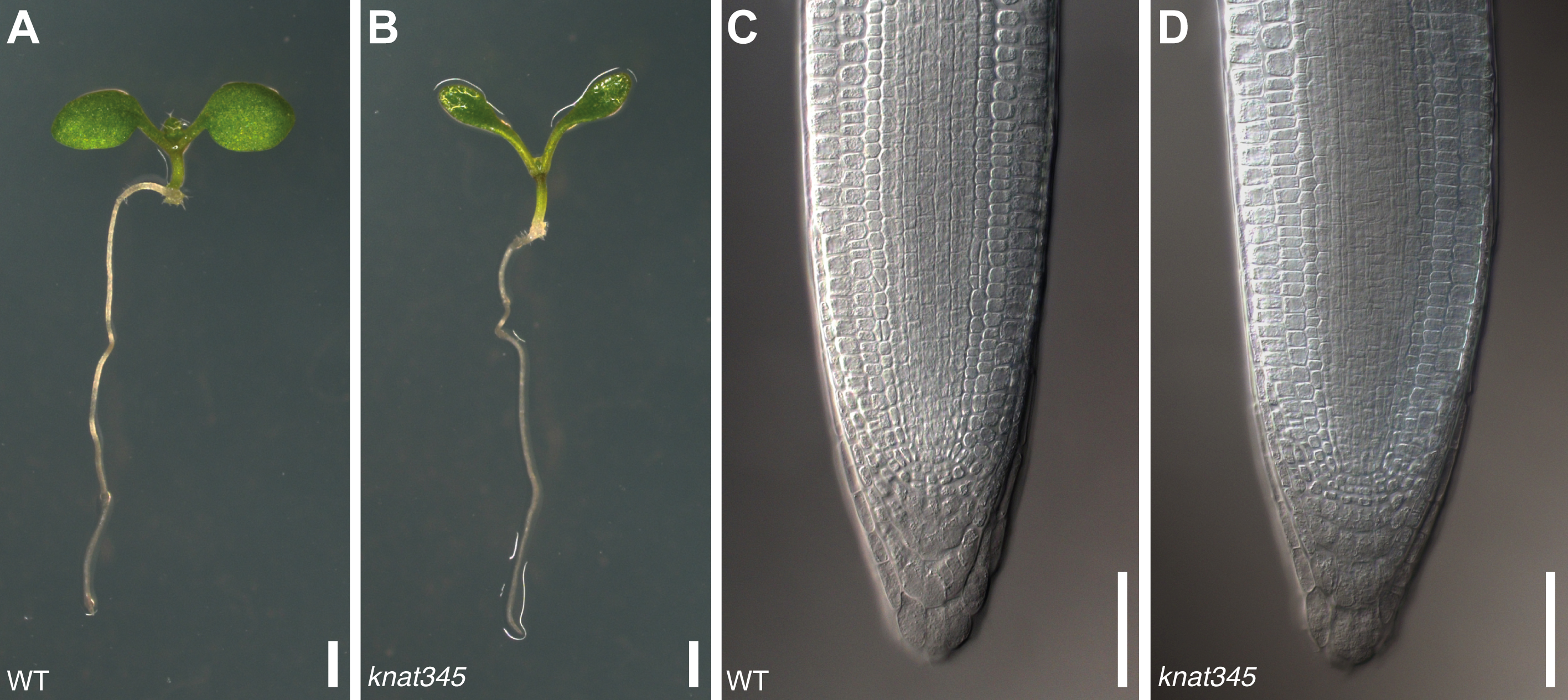

Supplement: S10 Fig — (A-B) 5-day-old wild-type (A) and knat345 (B) seedlings grown on nutrient agar plates. (C-D) DIC (differential interference contrast) optical sections through the root meristems of wild-type (C) and knat345 (D) plants. Plants are in the Col background. Scale bars in A, B, 1 mm and in C, D, 50 μm. (TIF) [file pgen.1004980.s010.tif]

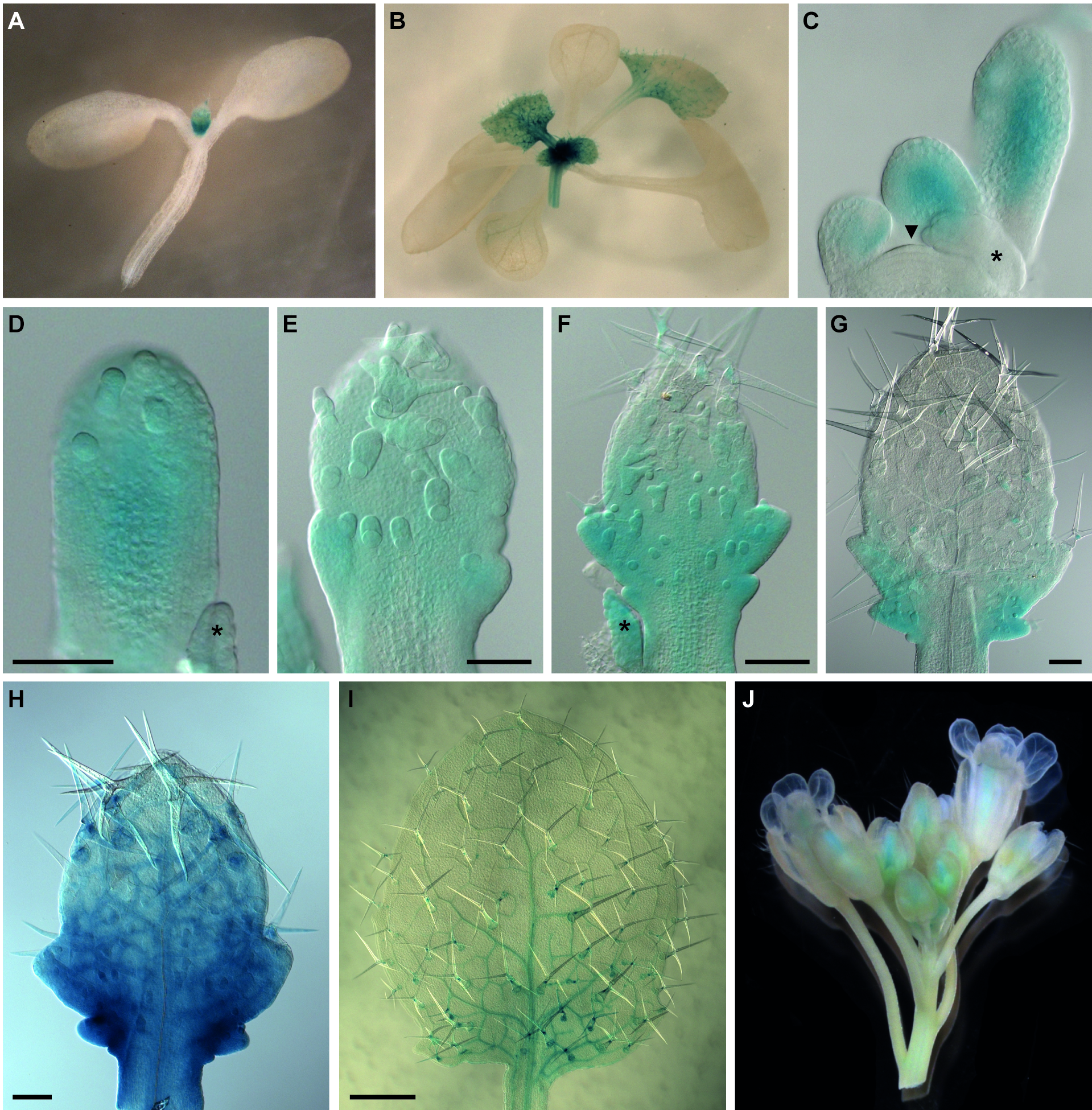

Supplement: S11 Fig — (A, B) pro KNAT5:KNAT5-GUS expression was detected in developing leaves. Reduced signal levels were observed in older leaves (B). (C) pro KNAT5:KNAT5-GUS activity is excluded from the shoot apical meristem (marked by an arrowhead). No detectable pro KNAT5:KNAT5-GUS signal was observed in the stipule (marked with an asterisk). (D-G) A series of leaves showing pro KNAT5:KNAT5-GUS expression from early (D) to late (G) stages of leaf development. Expression was first detected throughout leaf (D) and later becomes more restricted towards the proximal part of the lamina with strong expression in developing teeth (F-G). pro KNAT5:KNAT5-GUS expression was also detected in some stipules (marked with asterisks). (H-J) Prolonged, overnight incubation detected pro KNAT5:KNAT5-GUS along the vascular system in leaves (H-I) and gynoecia of flowers (J). (A-B, F, I) pro KNAT5:KNAT5-GUS expression was detected in the nucleus of trichomes. Plants are in the Col background. Scale bars in C-E, 50 μm and in I, 500 μm. (TIF) [file pgen.1004980.s011.tif]

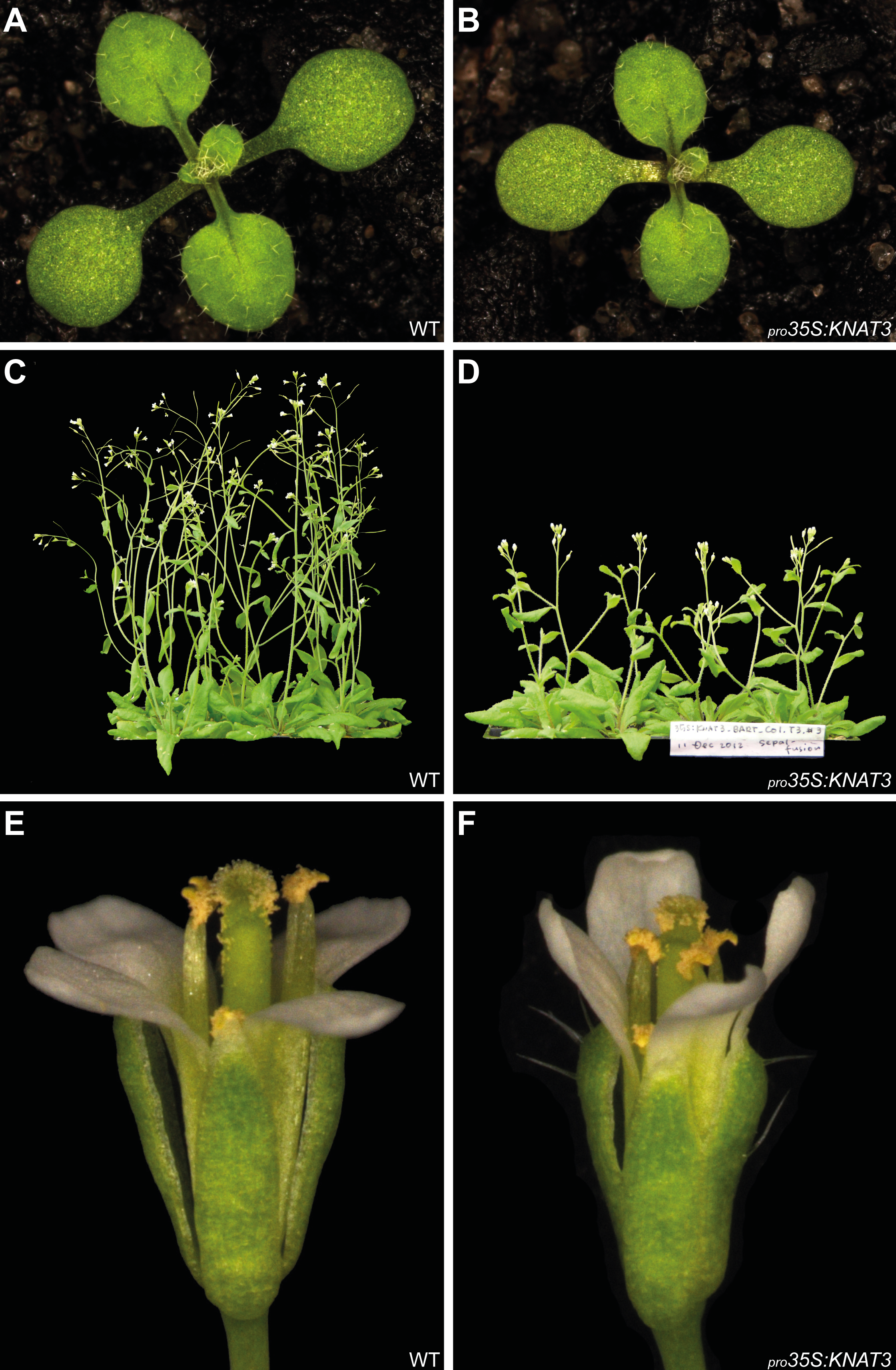

Supplement: S12 Fig — (A-D) Morphology of wild-type (A, C) and homozygous pro 35S:KNAT3 (B, D) plants. (A, B) Compared to wild-type (A), the shape of pro 35S:KNAT3 plants is more compact with shorter petioles and slightly smaller leaves (B). (C-D) The stems of pro 35S:KNAT3 plants (D) are shorter than those of wild-type (C). (E-F) Wild-type (E) and pro 35S:KNAT3 (F) flowers, showing fusion between sepals in pro 35S:KNAT3 flowers. Plants are in the Col background. Plants in (A, B), (C), and (D) are 12 days old, 6 weeks old, and 5 weeks old, respectively. (TIF) [file pgen.1004980.s012.tif]

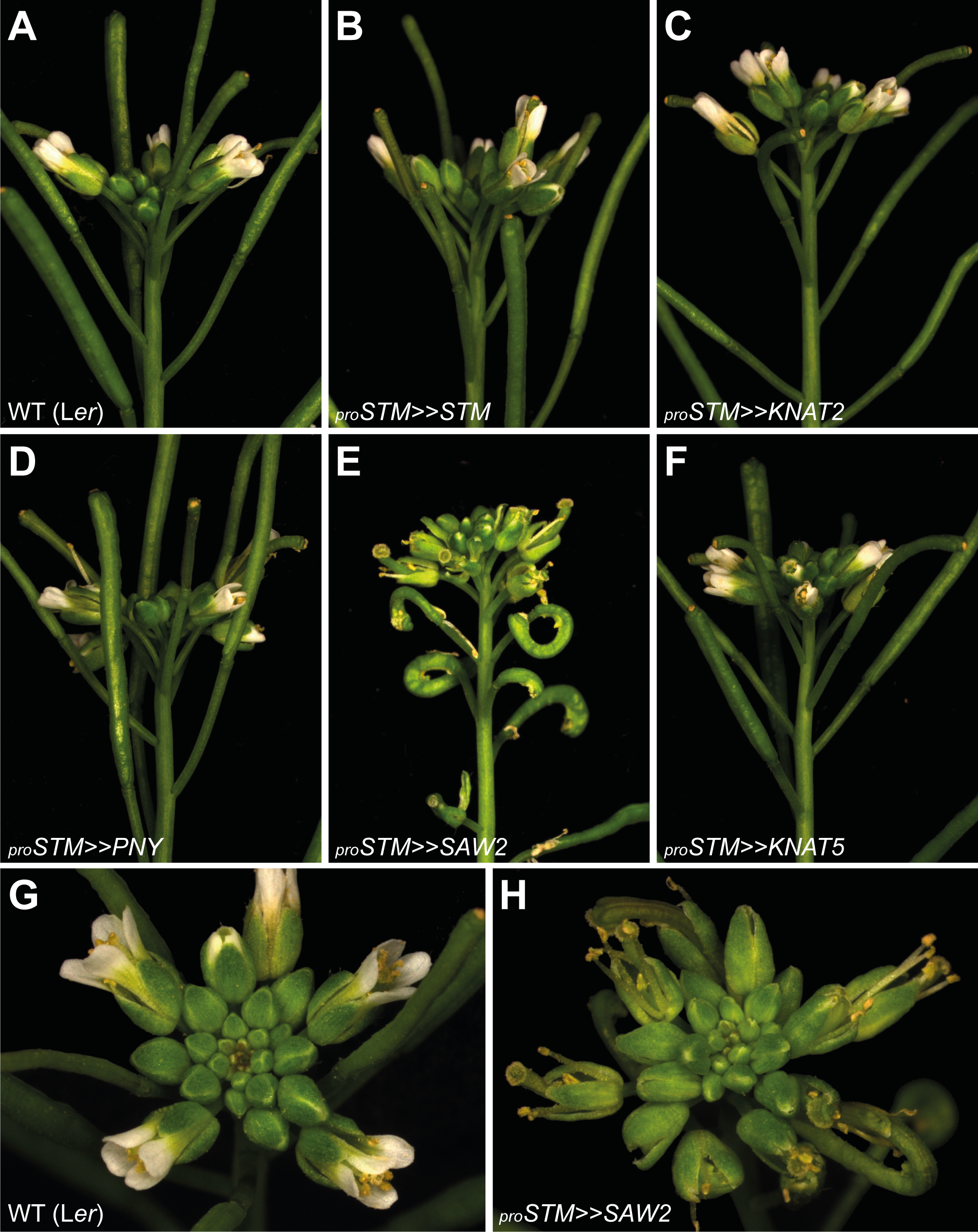

Supplement: S13 Fig — (A, G) Wild-type Ler inflorescences. (B-F, H) Inflorescences of transactivation lines expressing SAW2 (B, H), PNY (C), STM (D), KNAT2 (E), or KNAT5 (F) under the control of the STM regulatory sequence, pro STM. Expression of KNOX1 (e.g., STM and KNAT2), KNOX2 (e.g., KNAT5), or PNY does not impact flower development whereas abnormal phenotypes, such as fused sepals, reduced petals, and misshapen fruits, are observed in pro STM>>SAW2 flowers (B, H). Flower development was not impacted in pro STM>>KNAT5 plants, but fused sepals are observed in strong pro 35S:KNAT3 lines (S12F Fig.). Although studies based on the yeast two-hybrid technique suggest physical interactions between BELL and KNOX proteins in a rather nonspecific manner [29,32], the genetic data here and in Fig. 3 suggest KNOX2 proteins interact in planta with a subset of BELL proteins, including those of the BEL1/SAW1/SAW2 clade. KNOX1 proteins rely on a distinct set of BELL proteins, e.g. PNY and PNF [75,76]. Due to an obligate heterodimerization requirement, the activity of a KNOX/BELL pair may be limited by the protein with the more restricted expression domain. In Arabidopsis, KNOX2 functions appear to be regulated by restricted availability of corresponding BELL partners. Plants are in the Ler background. (TIF) [file pgen.1004980.s013.tif]

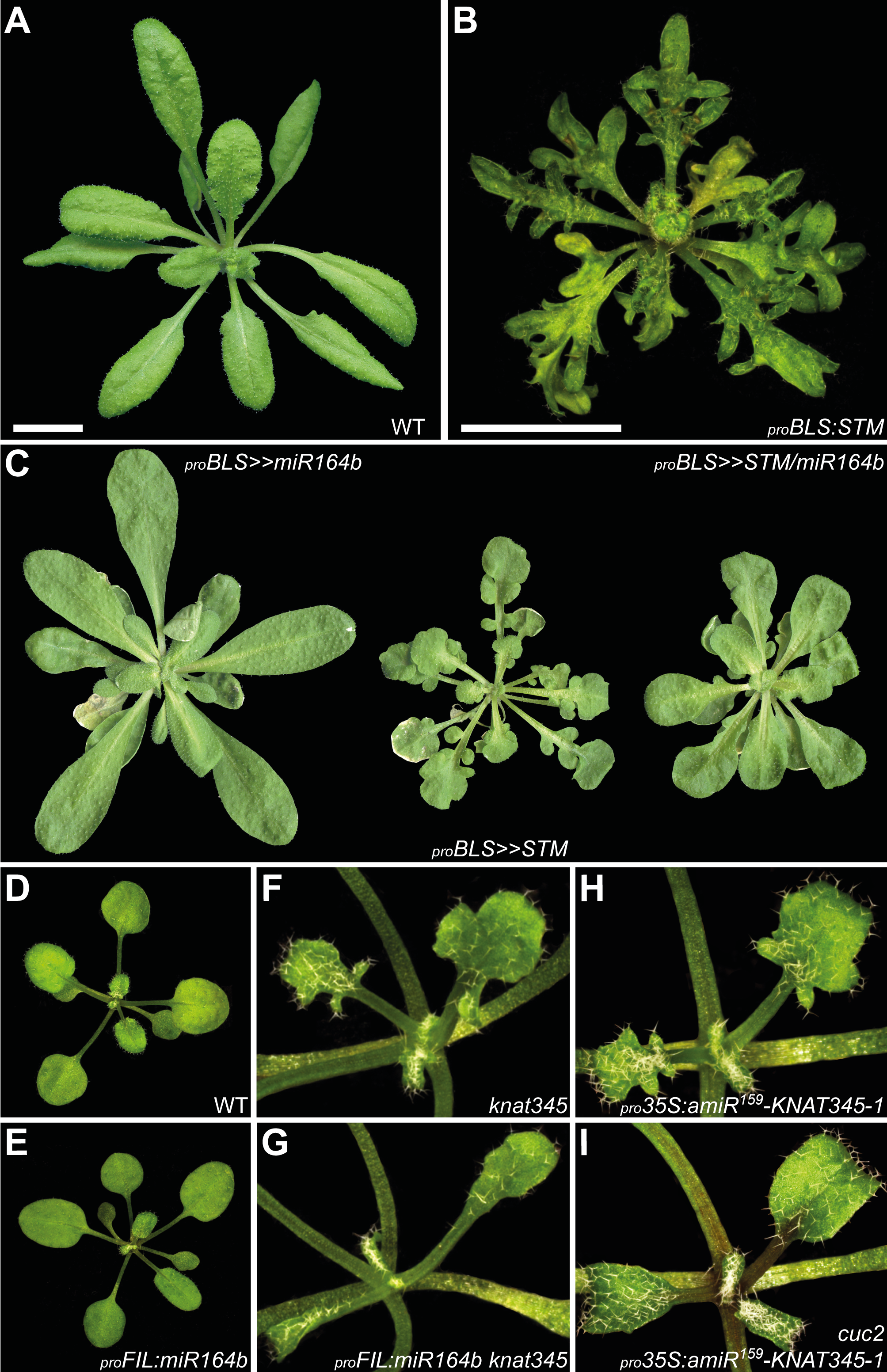

Supplement: S14 Fig — (A-B) Wild-type (A) and a plant with STM expression driven by the leaf specific promoter, pro BLS, pro BLS:STM (B), exhibiting a deeply lobed leaf phenotype characteristic of gain-of-function KNOX1 alleles. Plants are 6 weeks old. (C) From left to right are pro BLS>>miR164b, pro BLS>>STM, and pro BLS>>STM/miR164b plants, where either miR164b or STM alone, or both genes together, are transactivated by pro BLS. The miR164 family of miRNAs target CUC genes including CUC2, a key regulator of leaf serration. Leaf-specific miR164 expression largely suppresses serration development along the lamina margin both in wild-type and pro BLS>>STM backgrounds. Plants are grown at the same time under the short-day conditions. (D-E) Wild-type (D) and pro FIL:miR164b (E), where miR164b expression is driven in young leaves using regulatory sequences of the FILAMENTOUS FLOWER (FIL) gene (designated as pro FIL), plants. As in pro BLS>>miR164b plants, leaf serration is largely suppressed in pro FIL:miR164b plants. (F-G) pro FIL:miR164b suppresses the leaf serration phenotype of knat345 plants. Plants shown in (D-G) were grown for 25 days under short-day conditions. (H-I) Similarly, the cuc2 mutation largely suppresses the leaf serration phenotype of pro 35S: amiR 159 -KNAT345–1 plants. Close-ups of 7 weeks-old plants are shown. Plants in (C) are in the Ler background, and other plants are in the Col background. Scale bars in A, B, 1 cm. (TIF) [file pgen.1004980.s014.tif]

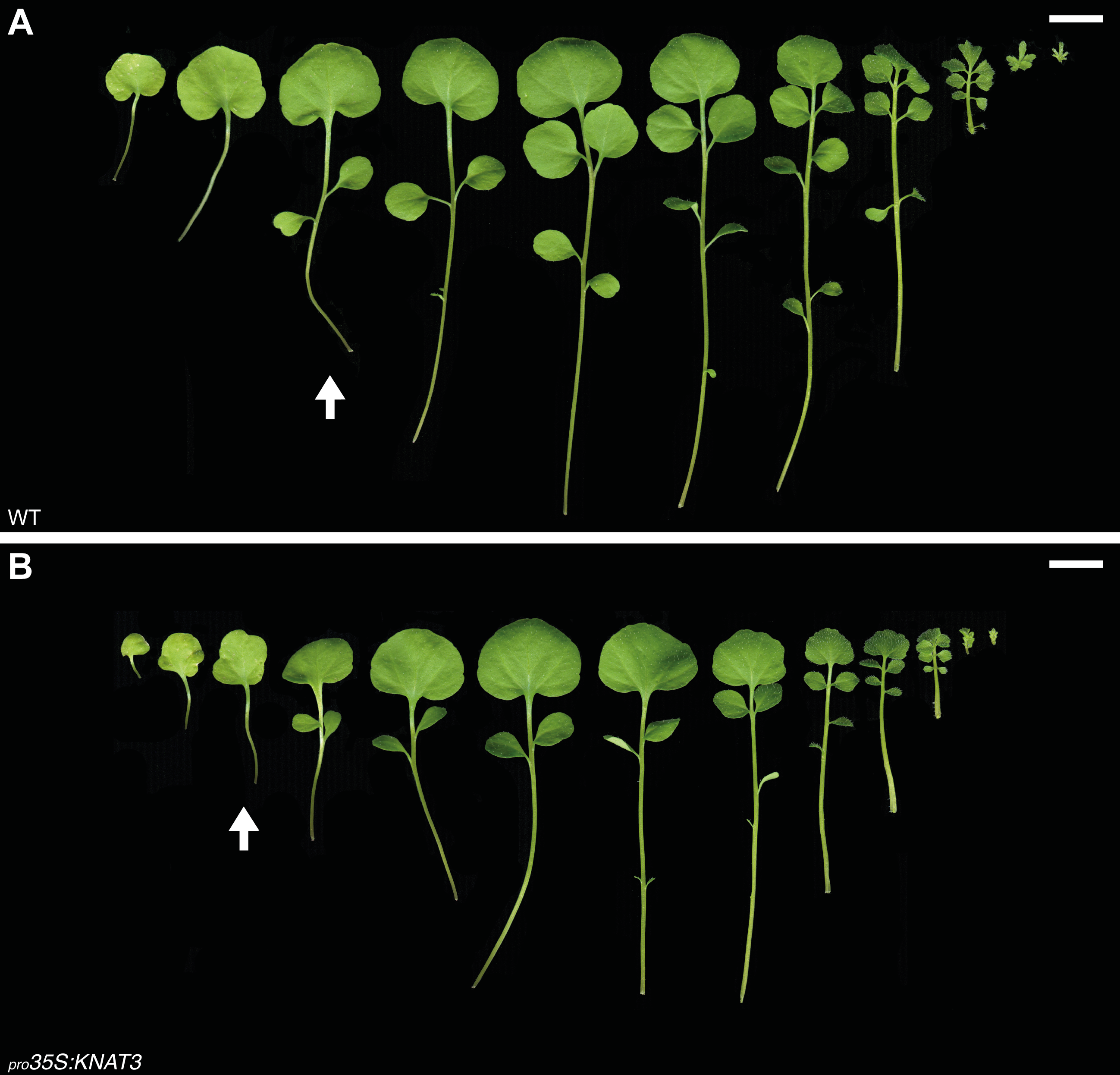

Supplement: S15 Fig — (A-B) Wild-type (A) and pro 35S:KNAT3 (B) leaves, removed from single plants and arranged in acropetal sequence (oldest to youngest) from left to right. In Cardamine hirsuta, leaf shape exhibits heteroblasty with leaflet number increasing in later produced leaves. Although leaflet number can vary for a particular leaf position, the first and second leaves always consist of a single, undivided, lamina, and the third leaf typically consists of three leaflets (marked by an arrow in A). (B) Gain-of-function KNOX2 allele (constitutive expression of the KNAT3 gene from Arabidopsis; pro 35S:KNAT3) in Cardamine hirsuta simplifies leaf shape, a phenotype particularly obvious in third leaves (indicated by arrows), which are undivided in strong lines. Plants are grown for one month. Scale bars, 1 mm. (TIF) [file pgen.1004980.s015.tif]

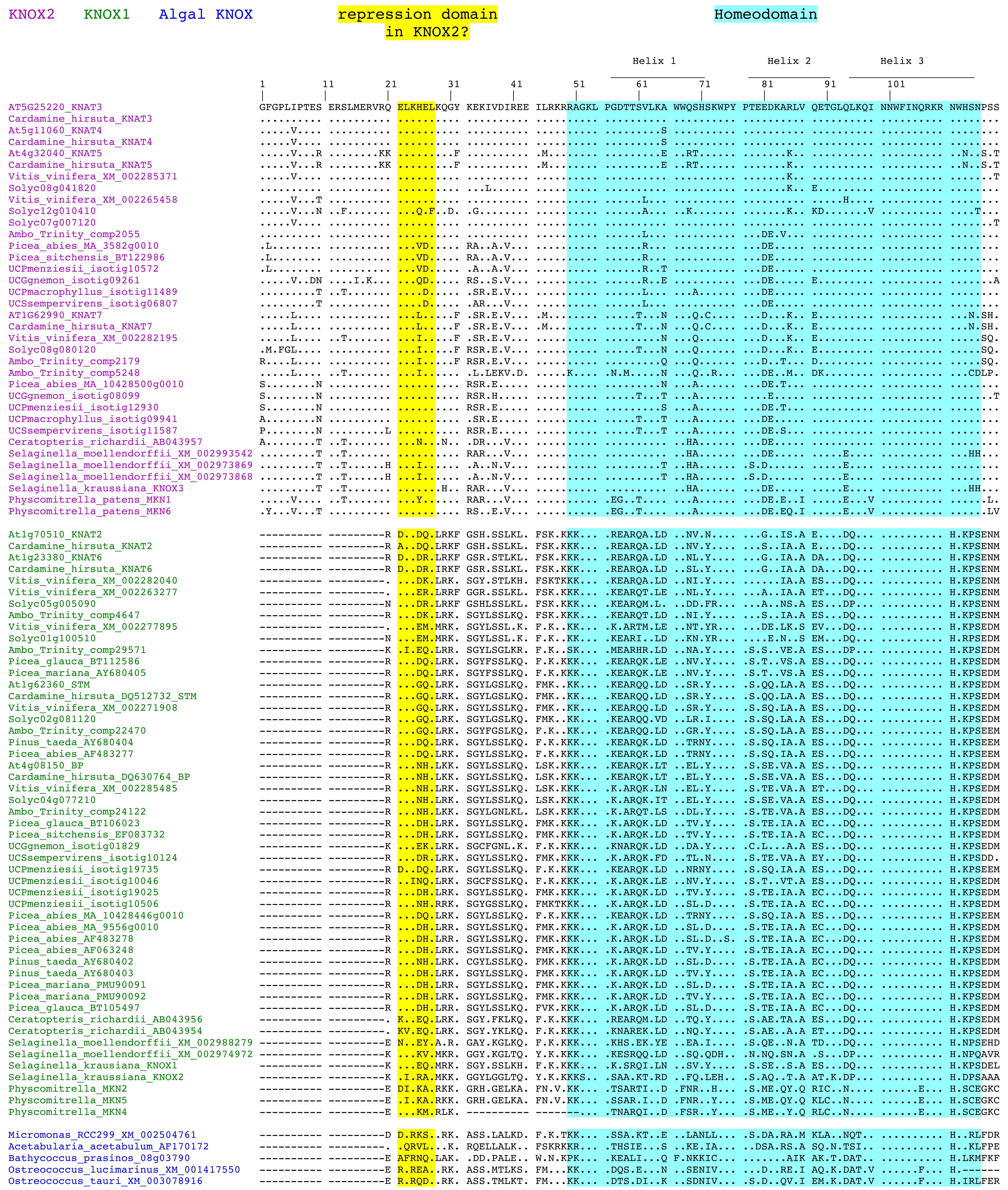

Supplement: S16 Fig — Amino acids identical to the one at the equivalent position in the Arabidopsis KNAT3 sequence are indicated with dots. Dashes denote a lack of corresponding sequence from the Arabidopsis KNAT3 sequence. Amino acid sequences that form three helices in the homeodomain are indicated. KNOX2, KNOX1, and algal KNOX sequences are color coded in magenta, green, and blue, respectively. The region encompassing the position of a presumptive KNOX2 repression motif (highlighted in yellow) and the homeodomain (highlighted in pale blue) is presented. The putative repression motif is absent in land plant KNOX1 and algal KNOX proteins and is one of the structural differences between KNOX1 and KNOX2 proteins [9]. Comparison of KNOX1 and KNOX2 homeodomains reveals that the third helices, an important determinant of DNA binding specificity, are highly conserved, indicating similar DNA binding properties, at least in vitro. (TIF) [file pgen.1004980.s016.tif]

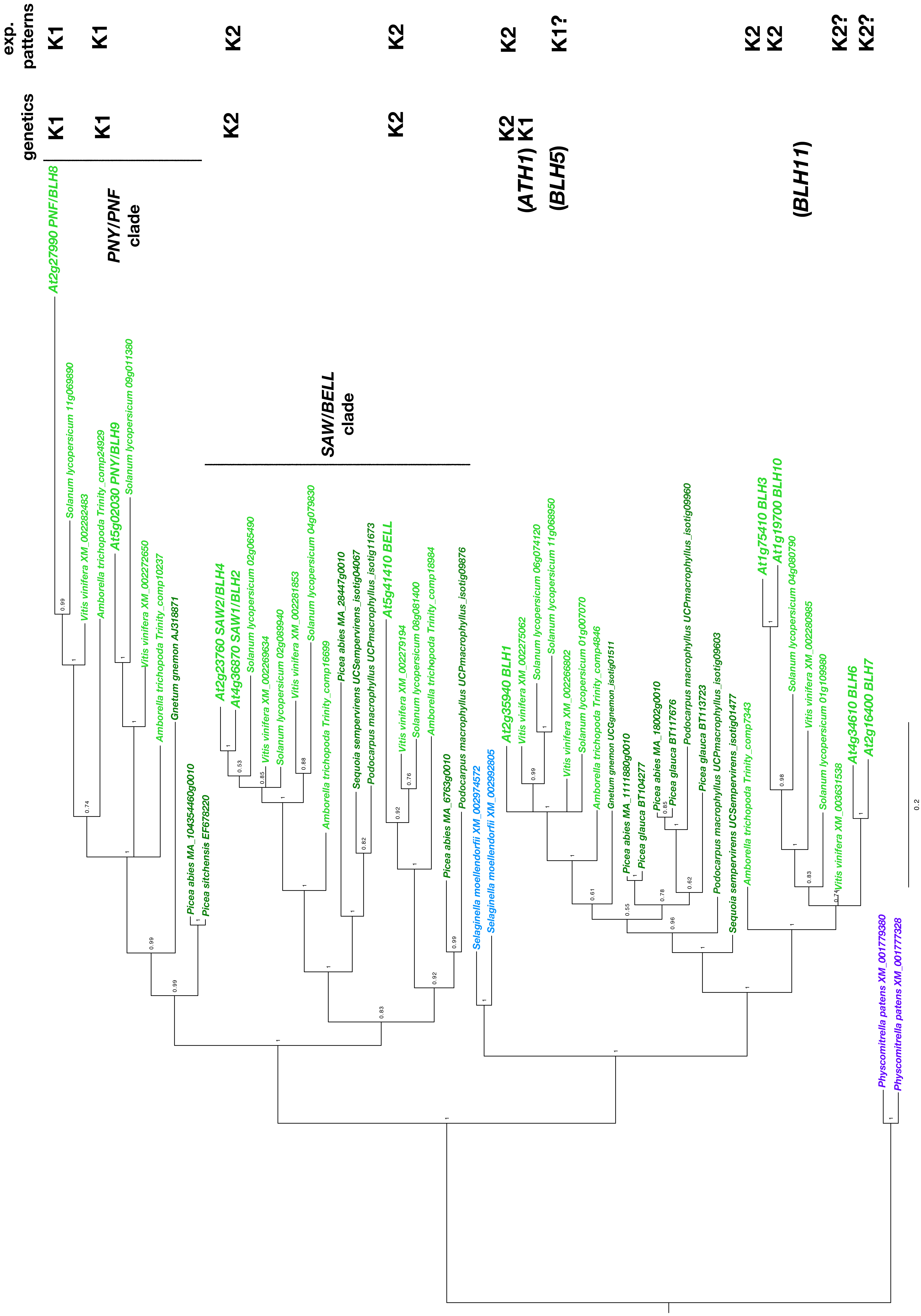

Supplement: S17 Fig — Numbers at branches indicate posterior probability values. Taxa are color coded according to major land plant clades: purple, moss; blue, lycophyte; dark green, gymnosperms; pale green, angiosperms. Clades that include Arabidopsis genes with known functions are indicated. Arabidopsis genes are highlighted using a larger font. Three Arabidopsis genes, ATH1, BLH5, and BLH11, were not included in this analysis because the sequences are divergent from those of other genes and cause long branch attraction and tree distortion. The approximate phylogenetic positions of these genes are indicated next to the phylogram with their names presented in parentheses. Based on genetic evidence (genetics) or overlapping expression patterns (exp. patterns) obtained from the previous and current studies, presumptive heterodimeric partners for Arabidopsis BELL proteins are postulated and placed beside the clades: K1 and K2 denoting KNOX1 and KNOX2 proteins, respectively. Ambiguous interactions are indicated by question marks. References for BELL-KNOX interactions are as per S3 Fig. Expression patterns were analyzed using ATTED-II (http://atted.jp/; [78]). Similar to KNOX genes, land plant BELL genes evolved from a single gene in the algal ancestor [8]. Note that the diversification of paralogs, however, followed a different trajectory in the two families as BELL genes do not fall into discrete functional clades. Namely, KNOX1-interacting BELL genes (PNY and PNF) form a sister clade with KNOX2-interacting BELL genes (BEL1 and SAW1/2), while genetic interactions implicate BLH1, from a phylogenetically distinct clade, as a KNOX2 partner since knat3 alleles suppress the phenotype induced by ectopic BLH1 expression [22,37]. (TIF) [file pgen.1004980.s017.tif]
