# Supplementary material for: Antagonistic Roles for KNOX1 and KNOX2 Genes in Patterning the Land Plant Body Plan Following an Ancient Gene Duplication
Source: PLoS Genet. 2015 Feb 11;11(2):e1004980. doi: 10.1371/journal.pgen.1004980 (PMC4335488; doi:10.1371/journal.pgen.1004980)
Supplement: S1 Table — (DOCX) [file pgen.1004980.s018.docx]

**S1 Table. Mutant and transgenic lines used in this study.**

| **Line** | **AGI code** | **Background** | **Source** |
| --- | --- | --- | --- |
| *knat3* | AT5G25220 | Columbia | ABRC (SALK_136464) |
| *knat4* | AT5G11060 | Columbia | NASC (N759461) |
| *knat5* | AT4G32040 | Columbia | ABRC (SALK_000339C) |
| *bp-9 knat2-5 knat6-1* | AT4G08150 (*BP*);  AT1G70510 (*KNAT2*);  AT1G23380 (*KNAT6*) | Columbia | [66] |
| *stm-11* | AT1G62360 | L*er* | [67] |
| *bel1-154* | AT5G41410 | Columbia | ABRC (CS871590) |
| *blh1-114* | AT5G41410 | Columbia | ABRC (CS410893) |
| *_pro_BP:GUS* | AT4G08150 | Columbia | [68] |
| *_pro_KNAT2:GUS* | AT1G70510 | C24 crossed into L*er* | [69] |
| *_pro_STM:GUS* | AT1G62360 | L*er* crossed into Columbia | Plasmid kindly provided from Miltos Tsiantis |
| *_pro_KNAT4:GUS* | AT5G11060 | Columbia | This study, Basta resistant |
| *KNAT5:GUS* | AT4G32040 | Columbia | This study, Basta resistant |
| *Op:KNAT2* | AT1G70510 | L*er* | [39] |
| *Op:STM* | AT1G62360 | L*er* | [39] |
| *Op:KNAT5* | AT4G32040 | L*er* | This study, Basta resistant |
| *Op:PNY* | AT5G02030 | L*er* | This study, Basta resistant |
| *Op:SAW2* | AT2G23760 | L*er* | This study, Kanamycin resistant |
| *_pro_STM:LhG4* | AT1G62360 | L*er* | [70] |
| *_pro_35S:KNAT3* | AT5G25220 | Columbia | This study, Basta resistant |
| *_pro_35S:KNAT3* | AT5G25220 | Cardamine | This study, Basta resistant |
| *_pro_BLS:STM* | AT1G62360 | Columbia | This study, Basta resistant |
| *_pro_35S:amiR^159^-KNAT4* | AT5G11060 | *knat3 knat5* | This study, Basta resistant |
| *_pro_35S:amiR^159^-KNAT345-1* | AT5G25220; AT5G11060; AT4G32040 | Columbia | This study, Basta resistant |
| *_pro_35S:amiR^159^-KNAT345-2* |  | Cardamine | This study, Basta resistant |
