# Supplementary material for: Antagonistic Roles for KNOX1 and KNOX2 Genes in Patterning the Land Plant Body Plan Following an Ancient Gene Duplication
Source: PLoS Genet. 2015 Feb 11;11(2):e1004980. doi: 10.1371/journal.pgen.1004980 (PMC4335488; doi:10.1371/journal.pgen.1004980)
Supplement: S2 Table — (DOCX) [file pgen.1004980.s019.docx]

**S2 Table. Primers used in this study.**

| **Primer name** | **Primer sequence (5’ to 3’)** | **Application** |
| --- | --- | --- |
| LBb1.3^a^ | ATTTTGCCGATTTCGGAAC | Detection of T-DNA |
| o3144^b^ | GTGGATTGATGTGATATCTCC | Detection of T-DNA |
| o8409^b^ | ATATTGACCATCATACTCATTGC | Detection of T-DNA |
| pCSA110LB-R | TACAGCAAGAACGGAATGCG | Detection of T-DNA |
| Knat3f | ATGGCGTTTCATCACAATCA | Genotyping (Knat3f/Knat3r for WT and Knat3f/LBb1.3 for *knat3*) |
| Knat3r | GGTAAGGCCATTTGGAATGA |  |
| Knat4f2 | GATCACCAAAAAGCTGGTACTC | Genotyping (Knat4f2/Knat4r3 for WT and Knat4r3/o8409 for *knat4*) |
| Knat4r3 | CATGAAGTGGTCAAGCTCCTTGTC |  |
| Knat5f | GACGGAGTCAACGCCGATGG | Genotyping (Knat5f/Knat5r for WT and Knat5r/LBb1.3 for *knat4*) |
| Knat5r | CCCACACTTGCTTAAGCGTTCG |  |
| KNAT3-XhoI-F | CTCGAGATGGCGTTTCATCACAATCATCTCTCACAAG | Coding sequence cloning |
| KNAT3-Bam/Xma-R | CCCGGGATCCTACGCGAACCGCTCTCTTCCGCTATTG | Coding sequence cloning |
| pKNAT4-PstI-F new | CTGCAGTGATGAGTGATCTCATATTAAGTTATGATTTCCGTC | _pro_KNAT4:GUS construct |
| pKNAT4-SalI-R new | GTCGACGTTTTCGTGTTGAATTTGTTTTTGAATTTCTGTTTCGG | _pro_KNAT4:GUS construct |
| knat5 5f spe | GATACTAGTTTAAAACCCTAGTTTCCGCTCAACTAATCC | KNAT5:GUS construct |
| knat5 5r kpn | CAGGTACCCGACTTCCCGGTCCTGAGTTTGGTTAC | KNAT5:GUS construct |
| knat5 3f pst | CATCTGCAGGTGACATAGCGGCTAACTAGAGGATGG | KNAT5:GUS construct |
| knat5 3r sal | CATGTCGACGTCATTAAGTCGGCATTCAGATTTTGGGCC | KNAT5:GUS construct |
| KNAT5 F | CTCGAGATGTCGTTTAACAGCTCCCA | Coding sequence cloning |
| KNAT5 R | GGATCCTACGACTTCCCGGTCCGTTT | Coding sequence cloning |
| BLH4/SAW2 F | AACTCGAGATGGGTTTAGCTACTACAACTTCTTC | Coding sequence cloning |
| BLH4/SAW2 R | TTGGATCCCTAAAAATCTCCAAAGTCTCTAACGGAG | Coding sequence cloning |
| At5g02030/PNY F | AACTCGAGATGGCTGATGCATACGAGCCTTATCATGTTC | Coding sequence cloning |
| At5g02030/PNY R | TTGGATCCTCAACCTACAAAATCATGTAGAAACTGATGA | Coding sequence cloning |
| BLH1-F | GATCTTTGAGTCTGACACAGAGACC | Genotyping (BLH1-F/BLH1-R for WT and BLH1-F/o8409 for *blh1-114*) |
| BLH1-R | ATCCGACGTGAATAGGCTGGTGTTG |  |

^a^The LBb1.3 primer is described in the SIGnAL website (http://signal.salk.edu/tdnaprimers.2.html).

^b^The o3144 and o8409 primer are described in the GABI-Kat website (http://www.gabi-kat.de/faq/vector-a-primer-info.html).
