# Supplementary material for: Antagonistic Roles for KNOX1 and KNOX2 Genes in Patterning the Land Plant Body Plan Following an Ancient Gene Duplication
Source: PLoS Genet. 2015 Feb 11;11(2):e1004980. doi: 10.1371/journal.pgen.1004980 (PMC4335488; doi:10.1371/journal.pgen.1004980)
Supplement: S3 Table — (DOCX) [file pgen.1004980.s020.docx]

**S3 Table. Primers used for semi-quantitative RT-PCR.**

| **Primer name** | **Primer sequence (5’ to 3’)** | **Target gene** |
| --- | --- | --- |
| Cyclophilin UP | TCTTCCTCTTCGGAGCCATA | Cyclophilin |
| Cyclophilin RP | AAGCTGGGAATGATTCGATG | Cyclophilin |
| KNAT3 UP | CAATCATCTCTCACAAGACCTCTCC | KNAT3 |
| KNAT3 RP | GGTAAGGCCATTTGGAATGA | KNAT3 |
| KNAT4 UP | ATGGCGTTTCATCACAATCA | KNAT4 |
| KNAT4 RP | GATCACCAAAAAGCTGGTACTC | KNAT4 |
| KNAT5 UP | GACGGAGTCAACGCCGATGG | KNAT5 |
| KNAT5 RP | CCCACACTTGCTTAAGCGTTCG | KNAT5 |
